# Genomic Features Predict Bacterial Life History Strategies in Soil, as Identified by Metagenomic Stable Isotope Probing

**DOI:** 10.1128/mbio.03584-22

**Published:** 2023-03-06

**Authors:** Samuel E. Barnett, Rob Egan, Brian Foster, Emiley A. Eloe-Fadrosh, Daniel H. Buckley

**Affiliations:** a School of Integrative Plant Science, Cornell University, Ithaca, New York, USA; b Department of Microbiology and Molecular Genetics, Michigan State University, East Lansing, Michigan, USA; c DOE Joint Genome Institute, Berkeley, California, USA; d Department of Microbiology, Cornell University, Ithaca, New York, USA; Oregon State University

**Keywords:** stable isotope probing, metagenome, soil, carbon cycle, microbial, genome, life history, microbial communities, microbial ecology

## Abstract

Bacteria catalyze the formation and destruction of soil organic matter, but the bacterial dynamics in soil that govern carbon (C) cycling are not well understood. Life history strategies explain the complex dynamics of bacterial populations and activities based on trade-offs in energy allocation to growth, resource acquisition, and survival. Such trade-offs influence the fate of soil C, but their genomic basis remains poorly characterized. We used multisubstrate metagenomic DNA stable isotope probing to link genomic features of bacteria to their C acquisition and growth dynamics. We identify several genomic features associated with patterns of bacterial C acquisition and growth, notably genomic investment in resource acquisition and regulatory flexibility. Moreover, we identify genomic trade-offs defined by numbers of transcription factors, membrane transporters, and secreted products, which match predictions from life history theory. We further show that genomic investment in resource acquisition and regulatory flexibility can predict bacterial ecological strategies in soil.

## INTRODUCTION

Soil-dwelling microorganisms are essential mediators of terrestrial C cycling (1–5), yet their immense diversity (6, 7) and physiological complexity, as well as the mazelike heterogeneity of their habitats (8–11), make it difficult to study their ecology *in situ*. A major limitation is that microbial contributions to soil C cycling cannot be defined on the basis of a discrete set of functional genes, such as for other biogeochemical processes (e.g., nitrification, denitrification, methanogenesis, methylotrophy, sulfate reduction, sulfide oxidation, etc.). The formation of persistent soil carbon, in particular, is largely governed by the formation of microbial macromolecules produced by anabolic processes. Life history theory has been proposed as a framework for predicting bacterial activity in soils (12–15). Life history theory proposes that fitness trade-offs define competitive interactions with respect to environmental characteristics (16, 17). These trade-offs are described in terms of energy allocation to growth, resource acquisition, and survival (3, 14, 18–20). For example, trade-offs between bacterial growth rate and yield are thought to constrain bacterial activity with respect to environmental variability (18). Such trade-offs can influence C fate by controlling the amount of C mineralized to CO_2_ or converted into microbial products that become soil organic matter (SOM) (18). Information about microbial ecological strategies can be used to improve the accuracy of global C-cycling models (21–23). Unfortunately, bacterial life history traits resist *in situ* characterization, and experiments with cultured strains often ignore the complex microbe-microbe and microbe-environment interactions that occur in soil (24).

In a previous study (15), we quantified the dynamics of C acquisition and growth for diverse soil-dwelling bacteria through multisubstrate DNA stable isotope probing (DNA-SIP) enabled by high-throughput 16S rRNA gene amplicon sequencing. This experiment tracked bacterial assimilation of nine different C sources through the soil food web over a period of 48 days (see Fig. S1 in the supplemental material). The nine C sources were selected to represent diverse molecules, which vary in bioavailability and are derived from plant biomass degradation. We defined bioavailability based on the ability of a molecule to cross the cell membrane and quantified it operational on the basis of solubility and hydrophobicity (15). Through this approach, we demonstrated that Grime’s C-S-R life history framework explains significant variation in bacterial growth and C acquisition dynamics in soil (15).

10.1128/mbio.03584-22.5 FIG S1 Experimental diagram of the previously described multisubstrate DNA-SIP study from Barnett et al. (15) (top) and the newly described metagenomic-SIP sequencing, processing, and analysis described here (bottom). In the top panel, the circles represent the days when microcosms were harvested, while the filled circles represent the samples chosen for metagenomic-SIP sequencing. The original DNA-SIP study used multiple-window high-resolution DNA-SIP to identify bacterial operational taxonomic units (OTUs) that assimilated ^13^C from each of the ^13^C sources within the harvested microcosms. The ^13^C-labeling patterns, along with population dynamics in soils (unfractionated DNA), were used to generate the three activity characteristics. To compare genomic features in ^13^C-labeled contigs within a treatment or MAG, we then averaged the characteristics of the OTUs either ^13^C labeled in the treatment or taxonomically mapped to the MAG and ^13^C labeled in the same treatment, respectively. For the metagenomic-SIP sequencing, pooled fractions between 1.72 and 1.77 g mL^−1^ were sequenced. ^13^C-labeled contigs were distinguished as being >1,000 bp long, having at least 5× coverage in the ^13^C treatment library, and having over a 1.5-fold increase in coverage in the ^13^C treatment compared to its corresponding ^12^C control. Download FIG S1, PDF file, 0.3 MB. Copyright © 2023 Barnett et al. 2023 Barnett et al. https://creativecommons.org/licenses/by/4.0/ This content is distributed under the terms of the Creative Commons Attribution 4.0 International license.

The C-S-R framework describes trade-offs with respect to resource acquisition and environmental variability (25, 26). Competitors (C) have high investment in resource acquisition and favor intermediate levels of environmental variability. Stress tolerators (S) have low investment in resource acquisition and are disfavored by temporal variability. Ruderals (R) have low investment in resource acquisition and are favored by high levels of temporal variability. Despite the growing interest in applying life history theory to explain bacterial activity and ecology in complex ecosystems, we know little of the genetic basis of life history traits. In this current study, we have sought to identify genomic features that underlie bacterial life history traits linked to the C-S-R framework.

Since the majority of soil-dwelling bacteria remain uncultivated and poorly described (27, 28), there is great utility in identifying genomic features that predict their ecological strategies (29). Genomic features of life history strategies have been identified in marine bacteria (30) and proposed for soil-dwelling bacteria (31). Genomic features associated with growth, resource acquisition, and survival are of particular interest when assessing life history trade-offs (13, 14, 25, 26). Numerous genes control such quantitative traits, however, and it is difficult to predict these complex traits *de novo* from genomic data. We hypothesized that life history strategies impose trade-offs that alter genomic investment in gene systems (i.e., numbers of genes devoted to a particular system) linked to resource acquisition (e.g., secreted enzyme production, secondary metabolite production, and membrane transport), environmental variability (e.g., transcriptional regulation, attachment, and motility), and survival (e.g., osmotic stress response and dormancy).

To link these gene systems to life history strategies, we performed metagenomic analysis of ^13^C-labeled DNA (metagenomic-SIP) produced in our previous multisubstrate DNA-SIP experiment (Fig. S1). The multisubstrate DNA-SIP experiment provided data on resource acquisition and growth dynamics for specific operational taxonomic units (OTUs; defined at 97% sequence identity; see Data Set S1). Metagenomic-SIP allowed us to map resource acquisition and growth dynamics onto ^13^C-labeled contigs and ^13^C-labeled metagenome-assembled genomes (MAGs). We developed several metrics for assessing resource acquisition and growth dynamics of bacterial taxa (15). Resource bioavailability was determined as the average bioavailability of the ^13^C-labeled C sources assimilated by an OTU. Maximum log_2_ fold change (max LFC) was determined as the maximal change in differential abundance of an OTU in response to C input. The latency of C assimilation was determined for OTUs as the difference in time between maximal ^13^C mineralization and earliest ^13^C labeling for a given C source. Latency changes in proportion to the likelihood that taxa engage in primary assimilation of ^13^C directly from a C source or secondary assimilation of ^13^C following microbial processing. For metagenomic-SIP, we selected eight of the ^13^C-labeled samples from the multisubstrate DNA-SIP experiment because these samples were enriched in genomes from taxa whose resource acquisition and growth dynamics represented extremes in the C-S-R life history framework (Fig. S1 and Fig. S2a). This strategy, by diminishing the confounding contribution of genomes from organisms having intermediate life history strategies, facilitates identification of genome features that underlie life history trade-offs. We took three approaches to analyzing these metagenomic-SIP data, each increasing in complexity, (i) a ^13^C-labeled contig-based approach to assess whether community-scale genome feature enrichment correlates with resource acquisition and growth parameters, (ii) a ^13^C-labeled MAG-based approach to assess whether genome feature enrichment correlates with resource acquisition and growth parameters, and (iii) a ^13^C-labeled MAG-based approach to assess trade-offs between genome features predicted from the C-S-R framework.

10.1128/mbio.03584-22.2 DATA SET S1 Data file (XLSX) that contains information on OTUs, MAGs, and metagenomic libraries as described in text. Download Data Set S1, XLSX file, 0.3 MB. Copyright © 2023 Barnett et al. 2023 Barnett et al. https://creativecommons.org/licenses/by/4.0/ This content is distributed under the terms of the Creative Commons Attribution 4.0 International license.

10.1128/mbio.03584-22.6 FIG S2 (A) Activity characteristic values of the ^13^C-labeled OTUs (as previously described in Barnett et al. [15]) detected in each ^13^C-labeled treatment subjected to metagenomic sequencing. Briefly, maximum log_2_ fold change represents the change in differential abundance from the initial condition (time zero) to the point when relative abundance was maximal for a given OTU. C source bioavailability is defined as the average bioavailability of all the substrates assimilated by each OTU, with the bioavailability of each C source defined operationally based on its mineralization dynamics (as previously described in Barnett et al. [15]). C assimilation latency is defined as the difference in time between the point when ^13^C source mineralization was maximal and the point at which an OTU was observed to assimilate the ^13^C substrate. (B) The number of genes in ^13^C-labeled contigs correlates with the number of ^13^C-labeled OTUs identified in Barnett et al. (15), suggesting that we recovered the genomic content of the target, active bacteria. Gene count is normalized by the number of reads recovered from the ^13^C treatment libraries (Table S1). Normalized number of genes found in the ^13^C-labeled contigs from each treatment. The numbers within or above the bars indicate the number of genes before normalization. (C) Relationship between the normalized number of genes in the ^13^C-labeled contigs and the number of OTUs ^13^C labeled under the same treatment (Pearson’s *r *=* *0.795, *P* = 0.018). The red line represents the linear regression, with red shading indicating the 95% confidence intervals. (D) Phylum-level breakdown of the taxonomically annotated genes in the ^13^C-labeled contigs under each treatment (genes) and the phylum-level breakdown of the ^13^C-labeled OTUs under each treatment in Barnett et al. (15) (OTUs). Genes used for this analysis were only those with taxonomic annotations. Gene taxonomy was assigned using the IMG pipeline. OTU taxonomy was assigned using the SILVA 111 release. (E) Abundance of genes from each feature in the ^13^C-labeled contigs from each treatment. For all features except SMBCs, feature abundance is calculated as the percentage of total protein-coding genes having the feature. For SMBCs, abundance is calculated as the number of SMBCs divided by the total protein-coding gene count. Download FIG S2, PDF file, 2.3 MB. Copyright © 2023 Barnett et al. 2023 Barnett et al. https://creativecommons.org/licenses/by/4.0/ This content is distributed under the terms of the Creative Commons Attribution 4.0 International license.

10.1128/mbio.03584-22.3 TABLE S1 Information on the metagenomic-SIP libraries. ^13^C-labeled contigs were defined as being over 1,000 bp long, having at least 5× coverage in the ^13^C treatment library, and having at least a 1.5-fold increase in coverage between the ^12^C control and ^13^C treatment libraries after accounting for sequencing depth. The gene counts include all genes predicted from the ^13^C-labeled contigs in each treatment. Download Table S1, PDF file, 0.10 MB. Copyright © 2023 Barnett et al. 2023 Barnett et al. https://creativecommons.org/licenses/by/4.0/ This content is distributed under the terms of the Creative Commons Attribution 4.0 International license.

The third approach was designed to identify bacterial life history strategies by characterizing trade-offs between genomic investment in regulatory flexibility and resource acquisition, as predicted from the C-S-R framework (25, 26). We chose to assess genomic investment in regulatory flexibility as the number of transcription factors (TF) relative to total gene number (TF/gene). Environmental variability will favor high TF/gene because transcription factors regulate gene expression in response to changes in the cellular environment (32). We chose to assess genomic investment in resource acquisition as the number of genes encoding secreted enzymes (SE), secondary metabolite biosynthetic pathways (SM), and membrane transporters (MT). Secreted enzymes and secondary metabolites (such as surfactants, siderophores, and antibiotics) enable bacteria to access and control extracellular resources that are otherwise unavailable for membrane transport because they are poorly soluble or sorbed to the soil matrix. Membrane transporters are required for uptake of substances available in the aqueous phase. Membrane transporter activity provides the physiological foundation for concepts of oligotrophy and copiotrophy, a life history framework used commonly to describe bacteria (12, 33–35). On the basis of previous conceptualizations of the C-S-R framework, we predicted a trade-off whereby investment in resource acquisition (SE + SM) would be highest relative to investment in membrane transport for intermediate levels of regulatory flexibility (TF/gene) and lowest at both high and low levels of regulatory flexibility. By clustering MAGs based on these trade-offs and comparing resource acquisition and growth parameters across clusters, we demonstrate the ability of these genomic features to predict bacterial life history strategies.

## RESULTS AND DISCUSSION

### Identification of ^13^C-labeled contigs with metagenomic-SIP.

We used metagenomic-SIP to enrich DNA from ^13^C-labeled bacteria and to identify ^13^C-labeled contigs, thereby linking genomic content to C acquisition. Overall, we recovered between 5 × 10^8^ and 1.3 × 10^9^ reads in each metagenome library after quality control (see Table S1 in the supplemental material). Coassembly generated over 1.2 × 10^6^ contigs that were >1,000 bp long, of which 639,258 were ^13^C labeled in at least one treatment (>5× coverage in the ^13^C treatment library and >1.5-fold enriched coverage relative to corresponding ^12^C controls [Table S1]). As expected, after normalizing for sequencing depth, the number of genes annotated from ^13^C-labeled contigs was positively correlated with the number of ^13^C-labeled OTUs in each treatment (Pearson’s *r *=* *0.795, *P *=* *0.018) (Fig. S2b and c). The phylum representation observed for ^13^C-labeled contigs differed somewhat from that observed for ^13^C-labeled OTUs as determined by 16S rRNA gene sequencing (Fig. S2d). This difference could be due to loss of some contigs from ^13^C-labeled metagenomic libraries on the basis of genome G+C content or due to differences in annotation methodologies used in metagenomic and 16S rRNA gene-based methods (Text S1).

10.1128/mbio.03584-22.1 TEXT S1 Supporting text and references as described in the text. Download Text S1, DOCX file, 0.04 MB. Copyright © 2023 Barnett et al. 2023 Barnett et al. https://creativecommons.org/licenses/by/4.0/ This content is distributed under the terms of the Creative Commons Attribution 4.0 International license.

### Genomic features of ^13^C contigs explain variation in resource acquisition and growth dynamics.

**FIG 1:**
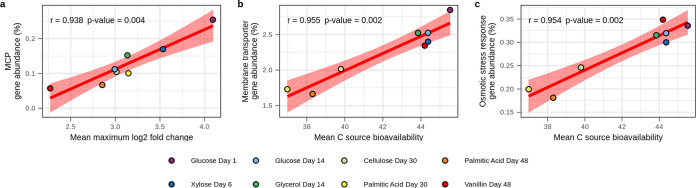
Genomic features of ^13^C-labeled contigs correlate with activity characteristics of ^13^C-labeled OTUs. (a) Abundance of methyl-accepting chemotaxis protein (MCP) genes correlates positively with the mean maximum log_2_ fold change (max LFC) of the ^13^C-labeled OTUs. (b) Abundance of membrane transporter genes correlates positively with the mean bioavailability of C sources acquired by the ^13^C-labeled OTUs. (c) Abundance of osmotic stress response genes correlates positively with the mean bioavailability of C sources acquired by the ^13^C-labeled OTUs. In all cases, the abundance is calculated as the percentage of protein-coding genes in ^13^C-labeled contigs that are annotated within the genomic feature. Red lines represent linear relationships, with shading indicating 95% confidence intervals. Pearson’s *r* and *P* values are provided. *P* values are adjusted for multiple comparisons using the Benjamini-Hochberg procedure (*n *=* *8).

We first tested whether the targeted genomic features explained variation in resource acquisition and growth dynamics at the community level, as assessed across the entire collection of ^13^C-labeled contigs (Fig. S2e; Fig. S3) and ^13^C-labeled OTUs observed from each ^13^C-labeled treatment (Data Set S1). This contig-based approach is meaningful because the ^13^C source identity had a large and significant effect on the identity of ^13^C-labeled taxa, with this variation driven by the overall dynamics of ^13^C assimilation and growth, as previously described (15). Three of the eight genomic features we examined explained significant variation in resource acquisition and growth dynamics (Fig. 1). Methyl-accepting chemotaxis protein genes (MCPs) were positively correlated with max LFC (Pearson’s *r* = 0.954, *P* = 0.002) (Fig. 1a), indicating that these genes are frequent in taxa that increase relative abundance dramatically in respond to new C inputs. In addition, membrane transporter (Pearson’s *r* = 0.907, *P* = 0.015) and osmotic stress response genes (Pearson’s *r* = 0.938, *P* = 0.004) were both positively correlated with C source bioavailability (Fig. 1b and c). Features that were not found to explain significant variation in the ^13^C-labeled contigs are discussed in Text S1.

10.1128/mbio.03584-22.7 FIG S3 Relationships between the frequency of 8 genome features in ^13^C-labeled contigs and all 3 *in situ* activity characteristics of ^13^C-labeled OTUs across treatments. For all except SMBCs, abundance is calculated as the percentage of protein-coding genes in ^13^C-labeled contigs that are annotated within the genomic feature. SMBC abundance is calculated as the SMBC count divided by total protein-coding gene count. Red or gray lines represent the linear relationships, with shading indicating the 95% confidence intervals. Red relationships are statistically significant, with *P* values adjusted for multiple comparisons using the Benjamini-Hochberg procedure (*n *=* *8). Correlation statistics are listed in Data Set S1. Download FIG S3, PDF file, 0.9 MB. Copyright © 2023 Barnett et al. 2023 Barnett et al. https://creativecommons.org/licenses/by/4.0/ This content is distributed under the terms of the Creative Commons Attribution 4.0 International license.

Soil consists of a complex matrix (36, 37) in which microbial access to C is limited by spatial and temporal variability (38, 39). Moisture is a major determinant of resource availability in soils, controlling soil matrix conductivity and tortuosity and thereby regulating rates of diffusion (40–43) as well as sorption/desorption kinetics (44). For these reasons, soil moisture is a major determinant of bacterial activity in soils (45–47). While resource concentration is a major determinant of bacterial growth kinetics in aquatic environments, bioavailability is a major determinant of bacterial growth kinetics in soil (15). Bioavailability, defined as the ability of a resource to cross the membrane, is determined in soil by solubility, sorption dynamics, and soil moisture (8, 48, 49). High-bioavailability C sources (e.g., glucose, xylose, and glycerol) are highly soluble, less likely to be sorbed to soil minerals, readily available for membrane transport, and their availability to cells governed primarily by diffusive transport as limited by soil moisture (35). These substrates are degraded rapidly, and so, elevated concentrations are ephemeral in soils (50). Hence, to compete effectively for highly bioavailable C sources, bacteria must exploit ephemeral periods when their resources are present in high concentrations. Low-bioavailability C sources (e.g., cellulose and palmitic acid), in contrast, cannot be transported directly across the membrane until they are transformed by extracellular microbial products such as secreted enzymes (3, 26, 51) or biosurfactants (52). These substrates are typically insoluble in soils and degraded over a span of weeks, months, or even years. Hence, to compete effectively for low-bioavailability C sources, soil-dwelling bacteria must invest in resource acquisition by manufacturing extracellular products that facilitate access to insoluble particulate materials.

Chemotactic bacteria can move through soil pore water and water films, allowing preferential access to C sources detected by MCPs (53, 54). MCPs are a dominant chemoreceptor family shared by diverse bacterial phyla (55, 56), and they are widely recognized as directing chemotaxis (56, 57). Our finding that MCP genes increase in proportion to the max LFC of bacterial taxa (Fig. 1a) suggests that chemotaxis is an important determinant of fitness for bacteria whose relative abundance increases dramatically during ephemeral periods of high resource availability. Similar explosive population dynamics are expected for organisms having a ruderal strategy as described in Grime’s C-S-R framework (25). Hence, we hypothesize that chemotaxis is adaptive in soils for growth-adapted bacteria that compete for ephemeral resources whose availability is driven by high environmental variability and that MCP gene count is a genomic feature that can help identify soil-dwelling bacteria having this life history trait.

Membrane transport regulates resource uptake, and transporter kinetics have been described as a key determinant of copiotrophic and oligotrophic life history strategies in aquatic environments (33–35, 58). Hence, membrane transport is likely a key determinant of bacterial life history strategies in soil. We show that high membrane transporter gene frequency correlates with the ability of soil bacteria to acquire high-bioavailability C sources (Fig. 1b). We hypothesize that high membrane transporter gene count is adaptive for bacteria that compete for ephemeral, highly bioavailable C sources. In soil, high membrane transporter gene count is likely indicative of more copiotrophic bacteria, with copiotrophs encompassing a wide diversity of life history strategies, including both ruderals and competitors as defined by Grime’s framework (25). We also hypothesize that low membrane transporter gene count is likely an indicator of oligotrophic bacteria that compete for less bioavailable C sources in soil, with low MT gene frequency indicating a tendency toward specialization in the resources used in diverse soil habitats.

Osmotic stress genes are affiliated with several cellular systems for surviving low water activity, including compatible solutes, aquaporins, and ion homeostasis (59, 60). Osmotic stress systems are of vital importance for microbial survival in soils due to the high variation in water activity (61, 62). We show that osmotic stress genes are more frequent in soil-dwelling bacteria that acquire C from highly bioavailable C sources (Fig. 1c). Highly bioavailable C sources are transiently abundant in water-filled pore space when soils are moist (63). Soil pores dry out rapidly as moisture becomes limiting; hence, we predict that osmotic stress is adaptive for bacteria that exploit resources present in water-filled pore space. In contrast, bacteria specializing in low-bioavailability C sources localize preferentially to surfaces (64). Water films and biofilms are favored on soil surfaces (42), buffering the organisms localized there from rapid variation in water activity. Our results suggest that osmotic stress is adaptive for soil-dwelling bacteria of more copiotrophic character (i.e., ruderals and competitors), and those that compete for high-bioavailability substrates whose availability corresponds with rapid changes in water activity.

One might naively predict that osmotic stress genes would be characteristic of organisms having a stress-tolerant life history strategy. The observation that osmotic stress genes do not predict a “stress-tolerant” bacterial lifestyle requires us to carefully consider how we define stress in bacterial ecology. Grime’s original framework, from plant ecology, describes plant stress as limitation for light, nutrients, and/or water, which are resources required for plant growth (25). This plant-centric definition of stress, based on resource limitation, conflicts with the microbiological definition in which stress is usually interpreted as abiotic stress (e.g., tolerance to pH, salinity, temperature, O_2_). Those bacteria that are adapted for resource limitation are typically defined as oligotrophs. Hence, Grime’s “stress tolerator” strategy, as interpreted in the proper ecological context, is indicative of bacteria having oligotrophic characteristics (15) and not those adapted for extremes of abiotic stress (e.g., extremophiles). These contrasting definitions of stress are a potential source of confusion when life history theory developed for plants is applied to bacteria. We propose that a better understanding of bacterial life history theory would be provided by interpreting the “S” in C-S-R as a scarcity adapted rather than stress adapted (15).

### Genomic features of ^13^C MAGs explain variation in resource acquisition and growth dynamics.

**FIG 2:**
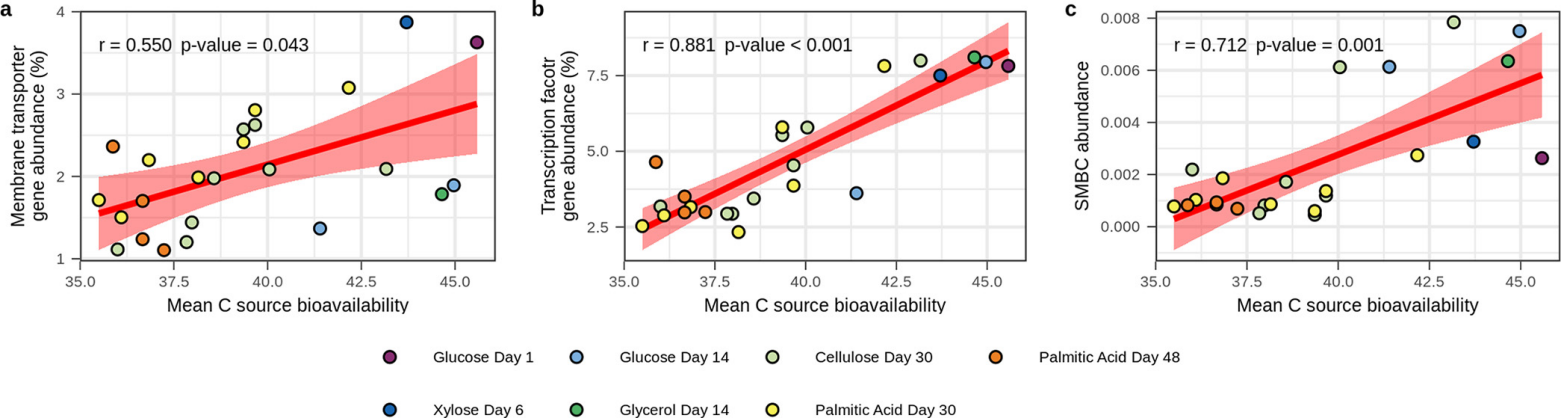
Genomic features of ^13^C-labeled MAGs correlate with activity characteristics of ^13^C-labeled OTUs taxonomically and isotopically mapped to MAGs. Membrane transporter gene frequency (a), transcription factor gene frequency (b), and secondary metabolite biosynthetic gene cluster (SMBC) abundance (c) all correlate positively with the mean bioavailability of C sources acquired. For membrane transporter and transcription factor genes, frequency is calculated as the percentage of protein-coding genes in MAGs that are annotated within the genomic feature. For SMBCs, abundance is the number of SMBCs divided by the number of protein-coding genes in MAGs. Red lines represent linear relationships, with shading indicating 95% confidence intervals. Pearson’s *r* and *P* values are provided. *P* values are adjusted for multiple comparisons using the Benjamini-Hochberg procedure (*n *=* *8).

A limitation of the contig-based analysis described above is that statistical power is low since we have only 8 treatments. Hence, we also used ^13^C MAGs to evaluate associations between genomic features and activity characteristics. We recovered 27 “medium-quality” MAGs (65) from the ^13^C-labeled contigs (>50% completeness and <10% contamination) (Data Set S1; Text S1). We linked these ^13^C-MAGs to corresponding OTUs that were ^13^C labeled in the exact same ^13^C-labeled DNA sample at the exact same time on the basis of taxonomic annotations (assigned by GTDB-tk [66]) (Data Set S1). For example, the ^13^C-labeled MAG Glucose_Day01_bin.1 was classified to the family *Burkholderiaceae* and therefore linked to all *Burkholderiaceae* OTUs ^13^C labeled in the glucose day 1 treatment. Three MAGs did not match any OTU (Cellulose_Day30_bin.7, PalmiticAcid_Day48_bin.4, and Vanillin_Day48_bin.1), and these unmatched MAGs were discarded from the analysis because growth and resource acquisition dynamics could not be assigned. While classification at the family level could group together functionally divergent taxa, the fact that these taxa were ^13^C labeled by the same substrates and at the same time in the same samples increases the likelihood that these groupings are ecologically meaningful. For each ^13^C-labeled MAG, activity characteristics were averaged across the matching ^13^C-labeled OTUs (Fig. S4; Data Set S1). We then evaluated the number of genes associated with each genomic feature, normalized for MAG size (Fig. S5; Data Set S1). As before, membrane transporter genes were positively correlated with C source bioavailability (Pearson’s *r* = 0.550, *P* = 0.043) (Fig. 2A), and we found that transcription factor genes (Pearson’s *r* = 0.881, *P* < 0.001) and secondary metabolite biosynthetic gene cluster (SMBC) abundance (Pearson’s *r* = 0.712, *P* = 0.001) were also positively correlated with C source bioavailability (Fig. 2b and c). Features that were not found to explain significant variation in the ^13^C MAGs are discussed in Text S1.

10.1128/mbio.03584-22.8 FIG S4 Activity characteristics of the ^13^C-labeled OTUs mapped to each ^13^C-labeled MAG. Boxplots are colored by the treatment under which ^13^C labeling occurs. Red bars indicate mean values. Three MAGs had no matching OTUs, Cellulose_Day30_bin.7, PalmiticAcid_Day48_bin.4, and Vanillin_Day48_bin.1. MAG and mapped OTU details are found in Data Set S1. Download FIG S4, PDF file, 0.7 MB. Copyright © 2023 Barnett et al. 2023 Barnett et al. https://creativecommons.org/licenses/by/4.0/ This content is distributed under the terms of the Creative Commons Attribution 4.0 International license.

10.1128/mbio.03584-22.9 FIG S5 Relationships between the abundance of all 8 genome features in ^13^C-labeled MAGs and all 3 activity characteristics of OTUs matching MAG taxonomy and ^13^C labeling. For all except SMBCs, abundance is calculated as the percentage of protein-coding genes in the MAG that are annotated within the genomic feature. SMBC abundance is calculated as the SMBC count divided by the total protein-coding gene count. Red or gray lines represent the linear relationships, with shading indicating the 95% confidence intervals. Red relationships are statistically significant, with *P* values adjusted for multiple comparisons using the Benjamini-Hochberg procedure (*n *=* *8). Correlation statistics are listed in Data Set S1. Download FIG S5, PDF file, 0.9 MB. Copyright © 2023 Barnett et al. 2023 Barnett et al. https://creativecommons.org/licenses/by/4.0/ This content is distributed under the terms of the Creative Commons Attribution 4.0 International license.

Having high numbers of transcription factors is thought to be an adaptive trait for microbes living in highly variable environments (32, 67, 68). Certain taxa are known to be enriched in transcription factor families, but the evolutionary basis of this variation in gene frequency is not well established (69). Our finding that transcription factor gene frequency correlates with C source bioavailability (Fig. 2b) suggests that growth on ephemeral C sources favors high transcription factor gene count because this adaptive trait allows bacteria to respond effectively to high environmental variability. The metabolic and physiological changes induced by these transcription factors may include previously discussed features such as MCP, membrane transporters, or osmotic stress systems. Our results support the idea that genomic investment in transcription factors is an adaptive trait that varies with environmental variability of the ecological niche.

Secondary metabolites include a wide range of small molecules produced by organisms. Bacteria often use these molecules to interact with their environments. Examples include antibiotics that kill or prevent the growth of other organisms, signaling molecules that mediate intercellular interactions, siderophores, chelators, and biosurfactants used to access insoluble nutrients (70). Secondary metabolites can facilitate competition for limited resources (71, 72), and they can even mediate microbial predation (73). Production of secondary metabolites requires multiple genes often found in clusters (i.e., SMBCs) (74, 75). We show that SMBC frequency correlates with C source bioavailability (Fig. 2c). This finding runs counter to the idea that secondary metabolites are important for competition for low-bioavailability resources (30, 70, 76). Given that this observation matches patterns observed for transcription factor and membrane transporter genes, we expect that SMBCs are favored by conditions of environmental variability and/or resource acquisition.

### Genomic feature correlation in publicly available soil genomes and metagenomes.

**FIG 3:**
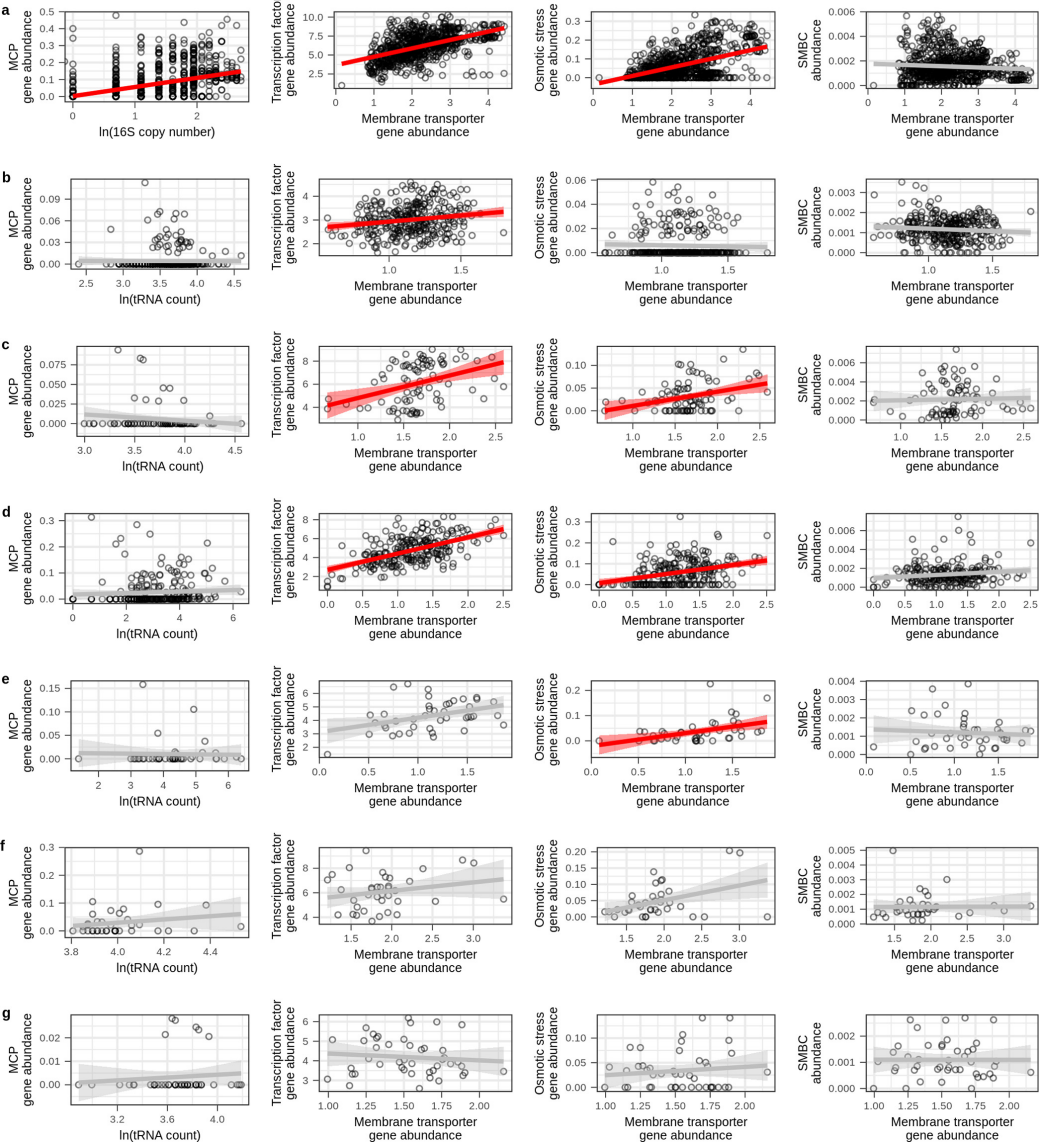
Membrane transporter gene frequency correlates with transcription factor and osmotic stress response gene frequencies in 4 of 7 independent metagenomic data sets examined, and MCP correlates with log *rrn* copy number in the RefSoil database. The tRNA gene count was used as a proxy for *rrn* copy number as described in text. The data sets are RefSoil genomes (a), Diamond et al. (101) MAGs recovered from drought-simulated meadow soils (b), Yu et al. (102) MAGs recovered from heavy DNA extracted from agricultural soils supplied with ^13^C-labeled ryegrass (c), Wilhelm et al. (103) MAGs recovered from heavy DNA extracted from forest soils treated with either ^13^C-labeled cellulose or lignin (d), Wilhelm et al. (104) phylobins recovered from heavy DNA fractions extracted from agricultural soil supplied with ^13^C-labeled cellulose (e), Zhalnina et al. (105) genomes isolated from Avena barbata rhizosphere (f), and Li et al. (106) MAGs recovered from rhizospheres of Zea mays, Triticum aestivum, and Arabidopsis thaliana (g). Red or gray lines represent the linear relationships, with shading indicating the 95% confidence intervals. Red relationships are statistically significant (adjusted *P* < 0.05), with *P* value adjusted for multiple comparisons within the data set using the Benjamini-Hochberg procedure (*n* = 4). Correlation statistics are in Data Set S1 in the supplemental material.

We observed through metagenomic-SIP that C source bioavailability correlates with membrane transporter gene, osmotic stress gene, transcription factor gene, and SMBC frequencies, and we hypothesize that these gene frequencies are predictive of an organism’s position on the copiotroph-oligotroph continuum. From this hypothesis, we predict that these genomic features should correlate in independent genomic and metagenomic data sets. We assessed these relationships in several data sets generated from a range of different soils (see Text S1). Since membrane transporter gene frequency was significantly associated with C source bioavailability at both the community level (^13^C-labeled contigs) and genome level (^13^C-labeled MAGs), we compared the gene frequencies for membrane transporter genes with those of transcription factor genes, osmotic stress genes, and SMBCs in each independent data set. Support for a relationship between membrane transporter and both transcription factor and osmotic stress genes was supported in 4 of 7 independent data sets (Fig. 3a to e). We found no correlation between membrane transporter genes and SMBC frequencies within any of the data sets (Fig. 3).

We also observed that MCP gene counts (Fig. 1a) and predicted rRNA gene (*rrn*) copy number (15) both correlate with max LFC when new C is added to soil. We hypothesize that these traits are linked to ruderal strategies (a subset of copiotrophs); hence, we predict that *rrn* copy number should correlate with MCP gene frequency in independent data sets. We compared MCP gene frequency to the natural log of either *rrn* copy number (for RefSoil) or tRNA gene count (for reference metagenome MAGs). While the RefSoil database contains complete genomes with accurate *rrn* copy numbers, MAGs from metagenomic data sets do not provide accurate *rrn* annotations; therefore, we used tRNA gene abundances as a proxy since tRNA gene count correlates with *rrn* copy number (77). In further support of this proxy, we observed that *rrn* copy number and tRNA gene count are strongly correlated in RefSoil bacterial genomes (Pearson’s *r* = 0.792, *P* < 0.001). The natural log of *rrn* copy number was positively correlated with MCP gene abundance across the RefSoil data set (Fig. 3a), yet the natural logs of the tRNA gene counts were not correlated with MCP gene abundance in any of the other data sets (Fig. 3b to g).

The correlational approach, as applied above, has two notable limitations. First, many of the genes in metagenomic data sets are poorly annotated. Inaccurate annotation can produce inaccurate gene counts for all of the gene systems we assessed. Second, adaptive trade-offs between gene systems will not produce straightforward correlations because the concept of a trade-off implies an interaction whereby the adaptive benefit varies depending on the life history strategy of the organism (78).

### Trade-offs in genomic investment define life history strategies.

Trade-offs occur when the benefit of a trait in a given environment differs between two groups. For example, increases in environmental variability might tend to favor more investment in resource acquisition for oligotrophic organisms (because an increase in resource variability in an environment lacking resources will tend to increase resource availability) but less investment in resource acquisition in copiotrophic organisms (because investing in extracellular products that enable resource acquisition provides little benefit in a highly disturbed environment). To detect such defining life history trade-offs among our ^13^C-labeled MAGs, we examined relationships between genomic investment in regulatory flexibility, as defined by transcription factor gene frequency (TF/gene); genomic investment in resource acquisition, as defined by the sum of gene counts for SE and secondary metabolite biosynthetic gene clusters (SM); and genomic investment in MT. We sum SM and SE because these features represent genomic investment in extracellular products used for resource acquisition. The products of extracellular reactions must undergo transport across the membrane prior to their metabolism; hence, we express genome investment in resource acquisition as the ratio (SE + SM)/MT. This ratio will be high for microbes producing numerous extracellular products and low for microbes that invest in uptake from the aqueous phase but are otherwise unable to acquire C from the soil matrix (which is mostly present in the particulate form or attached to soil minerals). Groups of genomes adapted to similar life history strategies should exhibit comparable genomic investment in these gene systems. We used *k*-means clustering based on TF/gene and (SE + SM)/MT to group all 27 MAGs into 3 clusters that we hypothesized would represent the C-S-R strategies. We then determined whether the properties of the genomes in each cluster matched predictions from the C-S-R framework.

**FIG 4:**
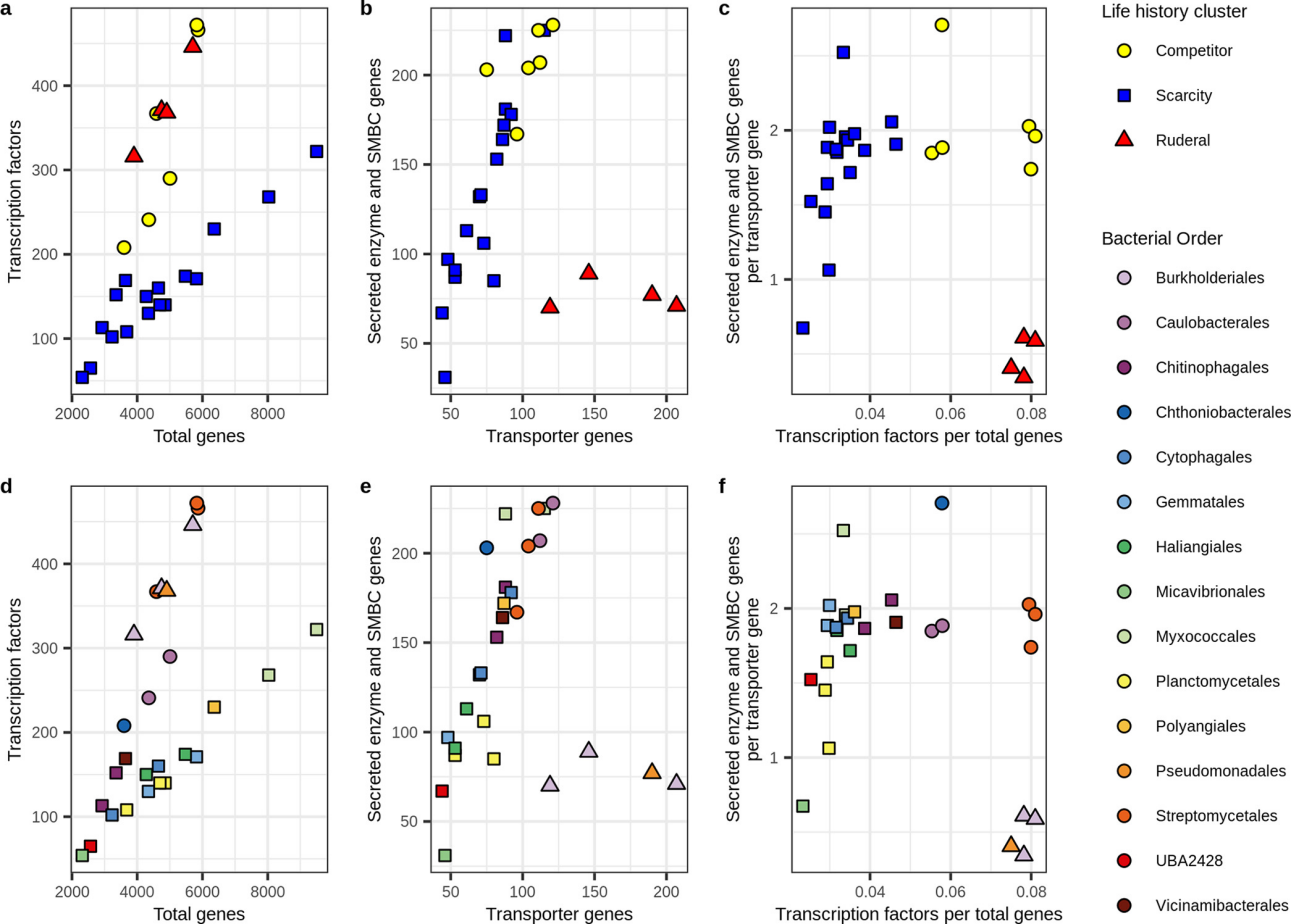
Genomic investment in gene systems can be used to cluster MAGs into life history strategies. (a) Relationship between transcription factor gene and total gene count. (b) Relationship between summed secreted enzyme and secondary metabolite biosynthesis gene counts and membrane transporter gene count. (c) Relationship between genomic investment in resource acquisition [(SE + SM)/MT] and regulatory flexibility (TF/genes). In this analysis, MAGs were clustered into life history strategies within the C-S-R framework using *k*-means clustering on scaled values of TF/genes and (SE + SM)/MT. Life history clusters are indicated by point colors in panels a to c. (d to f) The taxonomic identities of the MAGs (at the order level) corresponding to panels a to c.

We observed evidence for trade-offs in both regulatory flexibility and resource acquisition among these three clusters. Transcription factor genes tended to increase with total gene count (as expected), but TF/gene differed between the three clusters (Fig. 4a). When the genome size was small, the three clusters differed little in transcription factor gene count, but as total gene count increased, the clusters diverged, with one cluster having less regulatory flexibility than the other two (Fig. 4a). We also observe that SE + SM gene counts tend to increase in proportion to membrane transporter gene counts in two clusters (as expected since both are associated with extracellular resources), but the other cluster, which has the highest membrane transporter gene counts, maintains low SE + SM counts (Fig. 4b). When these relationships are plotted together, we observe that one cluster tends to increase relative investment in resource acquisition [(SE + SM)/MT] along with regulatory flexibility (TF/gene), while the other two have the opposite response (Fig. 4c).

These three clusters demonstrate adaptive trade-offs consistent with Grime’s C-S-R framework. The scarcity strategists (i.e., oligotrophs [S]) have low regulatory flexibility (Fig. 4a) and generally low genomic investment in transport (Fig. 4b), but their genomic investment in resource acquisition tends to increase in proportion to regulatory flexibility (Fig. 4c). That is, scarcity strategists whose ecological niches are the most constant require little genomic investment in regulatory flexibility and resource acquisition, while those whose niches are more variable require more investment in regulatory flexibility and resource acquisition. In contrast, ruderals (R) have high regulatory flexibility (Fig. 4a) and high investment in transport (Fig. 4b), but they have low genomic investment in resource acquisition (Fig. 4b and c). Finally, the competitive strategists (C) have intermediate to high levels of regulatory flexibility (Fig. 4a) and intermediate investment in membrane transport (Fig. 4b), but high genomic investment in resource acquisition (Fig. 4a), with little relationship between resource acquisition and regulatory flexibility (Fig. 4c). We expect many intermediate strategies among the C-S-R vertices, and as expected, we see that scarcity specialists adapted for high levels of regulatory flexibility are difficult to distinguish from competitive specialists adapted for lower levels of regulatory flexibility.

**FIG 5:**
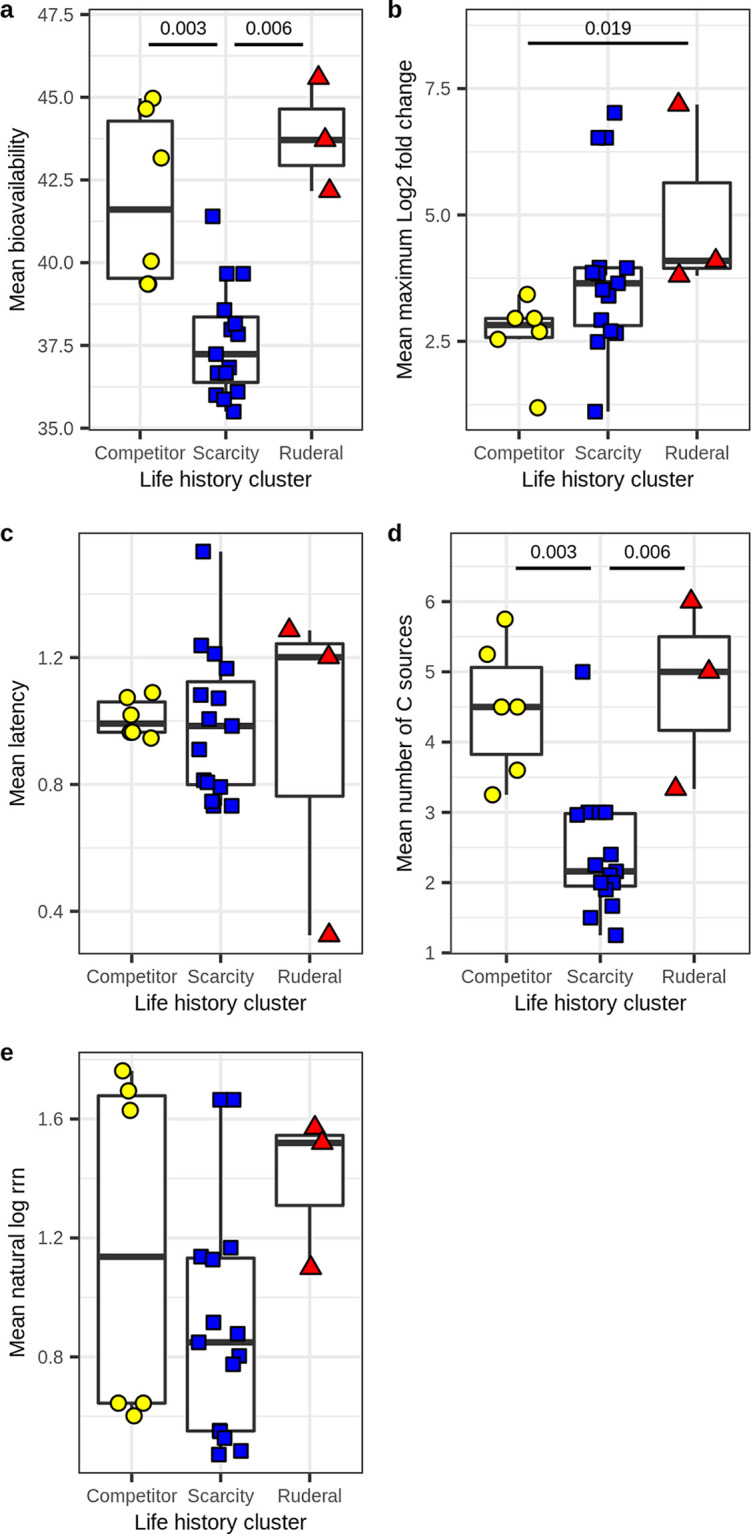
Resource acquisition and growth dynamics differ across life history strategies, indicative of trade-offs predicted from Grime’s C-S-R framework. Clusters corresponding to life history strategies were determined from *k*-means clustering based on TF/genes and (SE + SM)/MT, as previously indicated (from Fig. 4). Significance was determined by Kruskal-Wallis tests with *post hoc* comparisons performed using Dunn tests. (a) Bioavailability of ^13^C sources acquired was lower for scarcity-adapted MAGs than for competitor or ruderal MAGs. (b) Max LFC was higher for ruderal MAGs than competitor MAGs. (c) No difference was observed in latency across the three clusters. (d) The number of ^13^C sources acquired was lower for scarcity-adapted MAGs than for competitor or ruderal MAGs. (e) No difference was observed in the natural log of *rrn* copy number across the clusters.

MAGs assigned to the three clusters differ in their resource acquisition and growth dynamics, consistent with the expectations of life history theory. Ruderals and competitors acquired C sources that had significantly higher bioavailability than scarcity specialists (Fig. 5a), and they also consumed a higher diversity of C sources than the scarcity specialists; this difference was significant (Fig. 5d). Ruderals, however, had significantly higher max LFC relative to competitors, indicating the ability to increase population size dramatically in response to C input (Fig. 5b).

**FIG 6:**
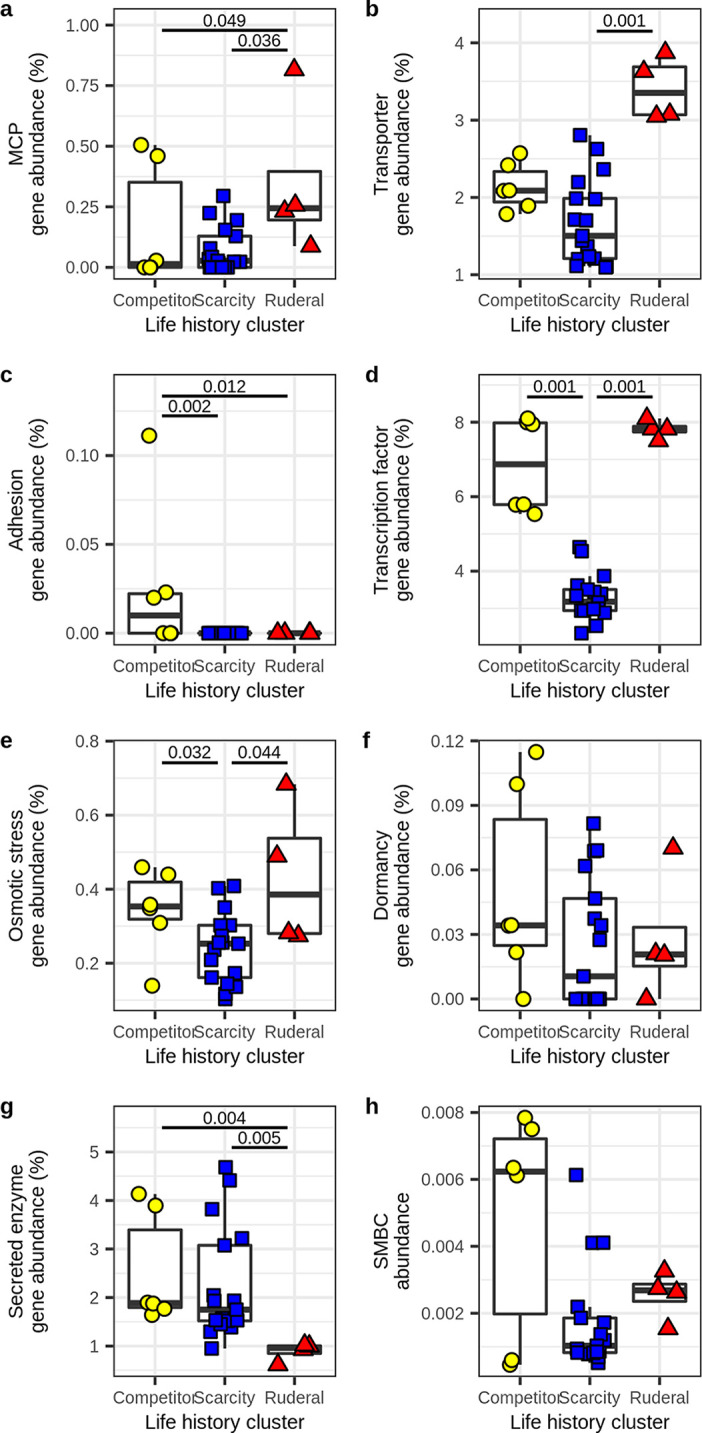
Genomic investment in gene systems differs across the three life history strategies, indicative of trade-offs predicted from Grime’s C-S-R framework. Clusters corresponding to life history strategies were determined from *k*-means clustering based on TF/genes and (SE + SM)/MT, as previously indicated (from Fig. 4). Significance was determined by Kruskal-Wallis tests with *post hoc* comparisons performed using Dunn tests. (a) Ruderal MAGs have a higher investment in MCP genes than competitor or scarcity-adapted MAGs. (b) Ruderal MAGs have a higher investment in membrane transporter genes than scarcity-adapted MAGs. (c) Competitor MAGs have a higher investment in adhesion genes than ruderal or scarcity-adapted MAGs. (d) Scarcity-adapted MAGs have a lower investment in transcription factor genes than ruderal or competitor MAGs. (e) Scarcity-adapted MAGs have a lower investment in osmotic stress response genes than ruderal or competitor MAGs. (f) There is no statistically significant difference in investment in dormancy genes across clusters. (g) Ruderal MAGs have a lower investment in secreted enzyme genes than competitor or scarcity-adapted MAGs. (h) There is no statistically significant difference in investment in SMBCs across life history clusters.

In terms of genomic features, we see that both ruderals and competitors have higher transcription factor and osmotic stress gene frequencies than scarcity specialists (Fig. 6a), while only the ruderals have higher membrane transporter gene abundance than scarcity specialists, and these differences are significant (Fig. 6a). Ruderals are distinguished from both competitors and scarcity specialists by their low investment in secreted enzymes and high investment in MCP (Fig. 6a). Competitors are distinguished from both scarcity and ruderal specialists by their higher investment in adhesion (Fig. 6a). The general theme is that both ruderals and competitors have copiotrophic characteristics, but ruderals appear to be opportunists with adaptations that maximize their ability to exploit ephemeral resources, while competitors have a greater genomic investment in resource acquisition. Scarcity specialists appear less well adapted for regulatory flexibility and more likely to specialize in their C sources (Fig. 5d). It is interesting to note that scarcity specialists did not have a high investment in adhesion genes despite their tendency to use C sources of low bioavailability. One hypothesis to explain this observation is that scarcity specialists exhibit metabolic dependency such that their access to insoluble C sources is facilitated by other members of the community. Support for this hypothesis comes from the fact that competitive specialists become labeled by low-bioavailability substrates after 7 to 14 days, while the scarcity specialists become labeled by these same substrates, but only after 14 to 48 days (15).

### Predicting ecological strategies from genome features.

**FIG 7:**
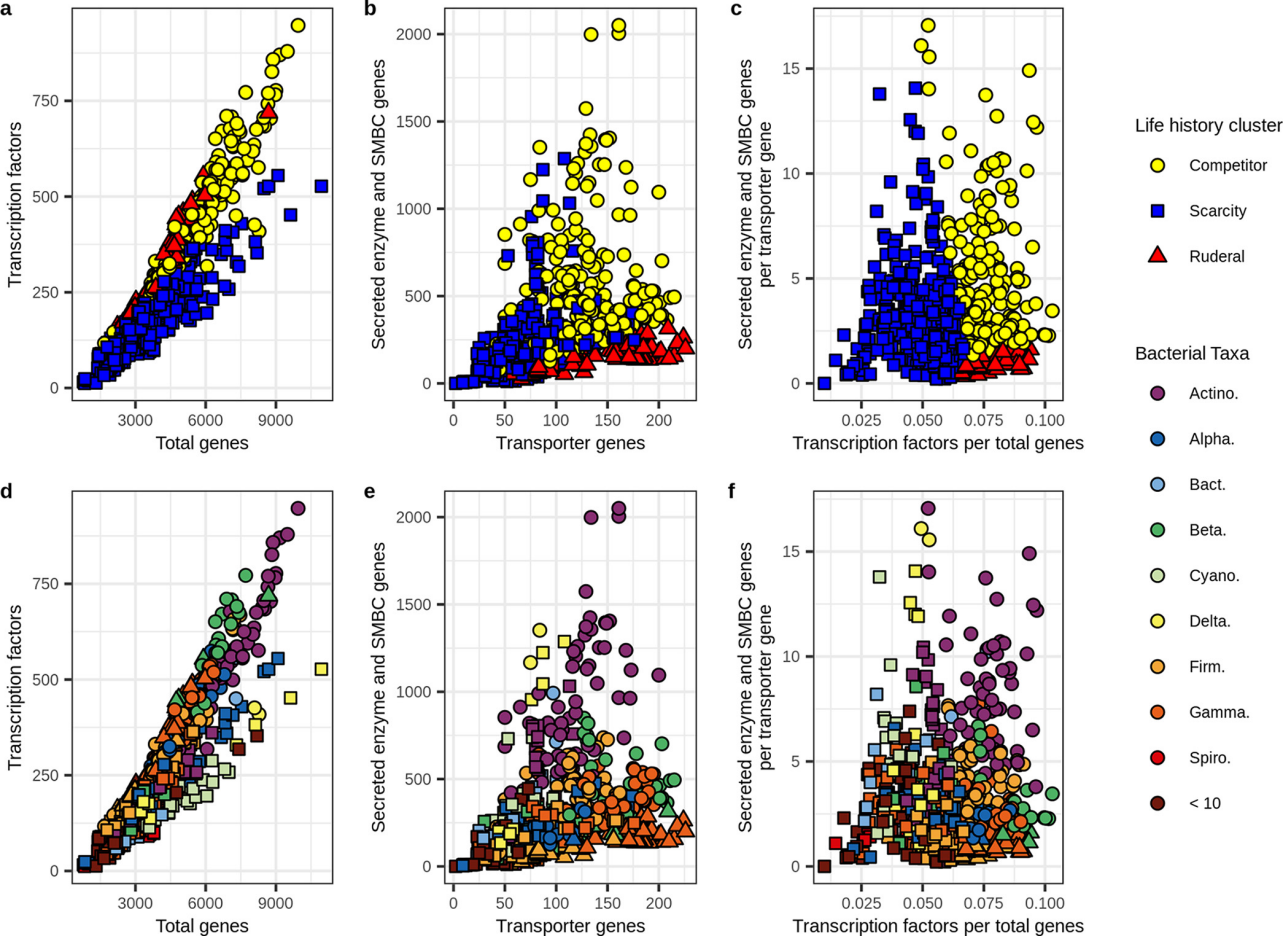
Trade-offs in genomic features can be used to predict life history strategies from reference genomes. RefSoil bacterial genomes were clustered based on genomic trade-offs between resource acquisition [(SE + SM)/MT] and regulatory flexibility (TF/genes) using *k*-means clustering trained by the clustering of the ^13^C-labeled MAGs (from Fig. 4). (a) Relationship between transcription factor gene count and total gene count. (b) Relationship between summed secreted enzyme and secondary metabolite biosynthesis gene counts and membrane transporter gene count. (c) Relationship between genomic investment in resource acquisition [(SE + SM)/MT] and regulatory flexibility (TF/genes). For panels a to c, clusters are colored by predicted life history strategies within the C-S-R framework. (d to f) Taxonomic identifies of genomes corresponding with panels a to c (at the phylum or class level; Actino., *Actinobacteria*; Alpha., *Alphaproteobacteria*; Bact., *Bacteroidetes*; Cyano., *Cyanobacteria*; Delta., Deltaproteobacteria; Firm., *Firmicutes*; Gamma., *Gammaproteobacteria*; Spiro., *Spirochetes*; and <10, aggregated taxa that have less than 10 genomes each).

We used parameters of TF/genjme and (SE + SM)/MT, defined from the three ^13^C-labeled MAG clusters described above, to predict life history strategies for RefSoil genomes. The resulting RefSoil genome clusters, predicted from these genome parameters, exhibited genomic characteristics representative of the expected life history trade-offs (Fig. 7a to c). The relationship between TF/gene and (SE + SM)/MT is roughly triangular, as we would expect for the C-S-R framework (Fig. 7c). Yet it is apparent that a vast diversity of intermediate life history strategies exist (Fig. 7c), and this is also an expected result since relatively few taxa will maximize adaptive trade-offs, while most will optimize adaptive traits to suit their particular ecological niche. Genomes having ruderal characteristics are enriched in the *Gammaproteobacteria* and *Firmicutes* (Fig. 7f; Fig. S6a), as we would expect, though members of these phyla can be found in all three clusters (Fig. S6b), owing to the vast diversity of these groups. In addition, genomes having competitive characteristics are highly enriched in the *Actinobacteria* and *Betaproteobacteria*, while genomes characteristic of scarcity specialists are enriched in the *Alphaproteobacteria* and other diverse phyla (e.g., *Verrucomicrobia*, *Acidobacteria*, *Gemmatimonadetes*, *Chloroflexi*, etc.) whose members are difficult to cultivate in laboratory media (Fig. 7f; Fig. S6a). Most bacterial phyla are metabolically and ecologically diverse, and we would not expect homogeneity among species within a phylum. In addition, previous observations show that C assimilation dynamics in soil are not well predicted by phylum-level classification (15). However, certain strategies are more common in some phyla than others, and these patterns, along with the taxonomic makeup of our MAG clusters (Fig. 4f), match general expectations. Furthermore, the three clusters we defined for RefSoil genomes possess patterns of genomic investment that match predictions derived from the C-S-R framework and are consistent with predictions based on the ^13^C-labeled MAGs (Fig. S6c; Table S2).

10.1128/mbio.03584-22.4 TABLE S2 Summary of parameter values for inferred life history clusters. A symbol (+) was added every time a cluster was observed to have a significantly higher value than another cluster in both ^13^C-MAG comparisons and the RefSoil comparisons. Abbreviations are defined in the text. Download Table S2, PDF file, 0.1 MB. Copyright © 2023 Barnett et al. 2023 Barnett et al. https://creativecommons.org/licenses/by/4.0/ This content is distributed under the terms of the Creative Commons Attribution 4.0 International license.

10.1128/mbio.03584-22.10 FIG S6 (A) Distribution of RefSoil bacterial taxa (at the phylum or class level) across the life history strategies predicted from clusters of TF/gene and (SE + SM)/MT. Percentage of genomes from each taxon in each predicted life history strategy with the total number of genomes used above each stacked bar. (B) Percentage of genomes in each predicted life history strategy cluster that are classified to each phylum/class. Phylum/class abbreviations are Actino., *Actinobacteria*; Alpha., *Alphaproteobacteria*; Bact., *Bacteroidetes*; Cyano., *Cyanobacteria*; Delta., Deltaproteobacteria; Firm., *Firmicutes*; Gamma., *Gammaproteobacteria*; Spiro., *Spirochetes*; and <10, taxa that contain less than 10 genomes. (C) Genomic investment in gene systems differs across life history strategies predicted from TF/genes and (SE + SM)/MT. Data are from RefSoil genomes with *k*-means clustering trained by clusters identified from ^13^C-labeled MAGs. In all cases, variation across life history clusters was first tested with Kruskal-Wallis tests, and, where statistically significant (*P* < 0.05), *post hoc* pairwise tests were performed using Dunn tests. Download FIG S6, PDF file, 0.7 MB. Copyright © 2023 Barnett et al. 2023 Barnett et al. https://creativecommons.org/licenses/by/4.0/ This content is distributed under the terms of the Creative Commons Attribution 4.0 International license.

### Conclusions.

Metagenomic-SIP enables us to link genome features to growth dynamics and C acquisition dynamics of bacteria as they occur in soil. We used a targeted approach, employing data from a multisubstrate DNA-SIP experiment, to select bacterial genomes that maximize life history trade-offs. We identified genomic features (MCP, membrane transporter genes, osmotic stress genes, transcription factor genes, and SMBCs) that are associated with growth and C acquisition dynamics of soil-dwelling bacteria. We also identified genomic signatures [TF/gene and (SE + SM)/MT] that represent life history parameters useful in inferring bacterial ecological strategies from genome sequence data. We show that while many intermediate strategies exist, there are diverse taxa that maximize life history trade-offs defined by these genomic parameters. The genomic signatures we identified are readily assessed using genomic and metagenomic sequencing, and these parameters may be useful in the assessment of bacterial life history strategies.

## MATERIALS AND METHODS

### Soil microcosms, DNA extraction, and isopycnic centrifugation.

The multisubstrate DNA-SIP experiment that provided the DNA samples we used for metagenomic-SIP has been described in detail elsewhere (15). An overview of the experimental design for this prior DNA-SIP experiment is provided for reference in Fig. S1 in the supplemental material. Briefly, a mixture of 9 different C sources was added to soil at 0.4 mg C g^−1 ^dry soil each (each representing about 3.3% of total soil C), moisture was maintained at 50% water-holding capacity, and sampling was performed destructively over a period of 48 days. All treatments were derived from the exact same soil sample (from an agricultural field managed under a diverse organic cropping rotation), they received the exact same C sources, and they were incubated under the exact same conditions; the only variable manipulated was the identity of the ^13^C-labeled C source. Eight ^13^C treatments from this prior experiment (each defined by the identity of the ^13^C source and the time of sampling) were chosen for metagenomic-SIP because the previous analysis (15) indicated that their ^13^C-labeled DNA was enriched in bacteria that maximized differences in life history strategy (Fig. S2a and see also Fig. 5e from the prior study [15]). The treatments selected for metagenomic-SIP were glucose, day 1; xylose, day 6; glucose, day 14; glycerol, day 14; cellulose, day 30; palmitic acid, day 30; palmitic acid, day 48; and vanillin, day 48. We also sampled ^12^C control treatments for days 1, 6, 14, 30, and 48 to facilitate identification of ^13^C-labeled contigs and improve metagenome assembly and binning (79). DNA used in this experiment (after undergoing extraction, isopycnic centrifugation, and fractionation) was the same as described previously (15) and was archived at −20°C for ~2 years prior to use in this study.

### Metagenomic sequencing.

For each of the eight treatments and five controls, we combined 10 μL of purified, desalted DNA solution from each CsCl gradient fraction having a buoyant density between 1.72 and 1.77 g mL^−1^. By pooling equal volumes from these fractions, we aimed to replicate the composition of the DNA pool of the entire heavy buoyant density window (1.72 to 1.77 g mL^−1^). Metagenomic-SIP simulations have demonstrated that this buoyant density range sufficiently enriches ^13^C-labeled bacterial DNA (79). DNA amplification and sequencing were performed by the Joint Genome Institute (JGI; Berkeley, CA, USA) using standard procedures. In short, DNA was amplified and tagged with Illumina adaptors using a Nextera XT kit (Illumina Inc, San Diego, CA, USA), and sequencing was performed on the NovaSeq system (Illumina Inc.).

### Read processing, metagenome assembly and annotation, and MAG binning.

Quality control read processing and contig assembly were performed by the JGI as previously described (80). Contigs were generated via terabase-scale metagenome coassembly from all 13 libraries using MetaHipMer (81). Gene calling and annotation of assembled contigs were performed through JGI’s Integrated Microbial Genomes and Microbiomes (IMG/M) system (82). Quality-filtered reads, coassembled contigs, and IMG annotations can be accessed through the JGI genome portal (CSP ID 503502, award at https://genome.jgi.doe.gov/portal/Micmetcarbocycle/Micmetcarbocycle.info.html). We mapped reads from each library to all contigs that were over 1,000 bp in length using BBMap (83) and then calculated contig coverages using jgi_summarize_bam_contig_depths from MetaBAT (84).

As we were primarily interested in genomes of bacteria that incorporated ^13^C into their DNA, we only used putatively ^13^C-labeled contigs to bin metagenome-assembled genomes (MAGs). Within each treatment, we defined a ^13^C-labeled contig as having an average read coverage greater than 5× in the ^13^C-treatment library and a 1.5-fold increase in coverage from the ^12^C control to ^13^C treatment library after accounting for the difference in sequencing depths. In calculating the fold increase in coverage, we normalized for sequencing depth by dividing coverage by read counts. We binned ^13^C-labeled contigs separately for each treatment based on both tetranucleotide frequency and differential coverage with MetaBAT2 (84), MaxBin (85), and CONCOCT (86). Default settings were used with the exceptions that minimum contig lengths were set to 1,000 bp for both MaxBin and CONCOCT and 1,500 bp for MetaBAT2. Final MAGs were generated by refining bins from all three binning tools using metaWRAP (87). Coverage information used during each binning run was from the paired ^13^C treatment and ^12^C control libraries, not the entire set of libraries. Therefore, we ran MAG binning eight separate times, once for each treatment. MAG qualities were calculated using CheckM (88). For further analyses, we only used MAGs with over 50% completeness and less than 10% contamination (i.e., “medium-quality” MAGs) following the guidelines for minimum information about metagenome-assembled genomes (65).

The binning approach we employed used coassembled contigs but binned these contigs separately across the eight ^13^C-labeled treatments. As such, some MAGs were identified in multiple treatments if their genomes were ^13^C labeled by multiple ^13^C-labeled C sources. These sister MAGs might represent a single population that can derive its C from multiple C sources or functionally distinct subpopulations each preferentially adapted for a different C source. Strain heterogeneity has previously been implicated as a cause of poor binning outcomes with soil metagenomes (89). Traditional MAGs tend to include the entire pan-genome of heterogeneous strains representing an individual taxon (90). Our ^13^C labeling-informed binning strategy should have a greater ability to differentiate functionally differentiated subpopulations than traditional binning strategies. Further characteristics of our MAGs are discussed in Text S1.

### Statistical analysis and computing.

Unless otherwise stated, all statistical analyses were performed and all figures generated with R (91) version 3.6.3. Code for all analyses and most processing is available through GitHub (https://github.com/seb369/CcycleGenomicFeatures).

### Testing associations between genomic features and activity characteristics.

We first assessed associations between genomic features and activity characteristics by comparing the genetic composition of ^13^C-labeled contigs with the averaged characteristics of the ^13^C-labeled OTUs identified in each corresponding treatment from our prior study (15). These OTUs were clustered at 97% sequence identity of 16S rRNA gene V4 region amplicons. We developed a list of eight genome features hypothesized to be associated with life history strategies and microbial C-cycling activity in soil environments as follows. (i) MCP genes were identified by the product name “methyl-accepting chemotaxis protein.” (ii) Transporter genes were identified by product names containing the terms “transporter,” “channel,” “exchanger,” “symporter,” “antiporter,” “exporter,” “importer,” “ATPase,” or “pump.” The resulting gene list was then filtered to include only those predicted by TMHMM (92) (version 2.0c) to have at least one transmembrane helix. (iii) Adhesion-associated genes included adhesins and holdfast and identified by product names “holdfast attachment protein HfaA,” “curli production assembly/transport component CsgG/holdfast attachment protein HfaB,” “adhesin/invasin,” “fibronectin-binding autotransporter adhesin,” “surface adhesion protein,” “autotransporter adhesin,” “adhesin HecA-like repeat protein,” “ABC-type Zn^2+^ transport system substrate-binding protein/surface adhesin,” “large exoprotein involved in heme utilization and adhesion,” “Tfp pilus tip-associated adhesin PilY1,” and “type V secretory pathway adhesin AidA.” (iv) Transcription factor genes were first identified by product names containing the terms “transcriptional regulator,” “transcriptional repressor,” “transcriptional activator,” “transcription factor,” “transcriptional regulation,” “transcription regulator,” or “transcriptional [family] regulator,” where “[family]” is replaced by some gene family identification. Additional transcription factor genes were identified from the protein FASTA sequences using DeepTFactor (93). (v) Osmotic stress-related genes were identified by product names containing the terms “osmoregulated,” “osmoprotectant,” “osmotically inducible,” “osmo-dependent,” “osmolarity sensor,” “ompr,” and “l-ectoine synthase.” (vi) Dormancy-related genes covered three different mechanisms (94). Endospore production was indicated by products containing the name “Spo0A,” though no Spo0A genes were found. Dormancy resuscitation was indicated by products containing the name “RpfC,” a resuscitation-promoting factor. Dormancy-related toxin-antitoxin systems were indicated by products containing the names “HipA,” “HipB,” “mRNA interferase MazF,” “antitoxin MazE,” “MazEF,” “RelB,” “RelE,” “RelBE,” “DinJ,” or “YafQ.” (vii) Secreted enzyme genes were first annotated against three enzyme databases to include enzymes important for breakdown of organic matter. Carbohydrate-active enzymes were annotated by mapping protein sequences to the dbCAN (95) database (release 9.0) with HMMER using default settings. Of these enzyme genes, only those in the glycoside hydrolase (GH), polysaccharide lyase (PL), or carbohydrate lyase (CE) groups were retained. Proteases were annotated by mapping protein sequences to the MEROPS (96) database (release 12.3) using DIAMOND BLASTP alignment with default settings except an E value of <1 × 10^−10^. Enzymes containing an α/β hydrolysis unit were annotated by mapping protein sequences to the ESTHER (97) database (downloaded 11 June 2021) with HMMER using default settings. While some enzymes containing α/β hydrolysis units are included in the carbohydrate-active enzymes, this group also includes lipases. All annotated enzyme genes from these three groups were then filtered to those containing a secretion signal peptide sequence annotated by SignalP (98) (version 5.0b). Gram-positive annotations were used for any genes annotated to the *Firmicutes* or *Actinobacteria* phyla, and Gram-negative annotations were used for all others. (viii) Bacterial secondary metabolite biosynthetic gene clusters (SMBCs) were predicted using antiSMASH (99) (version 5.1.2) with default settings.

For each genomic feature, except for SMBCs, we calculated the percentage of all protein-coding genes from each ^13^C-labeled contig pool (i.e., ^13^C labeled in each treatment) that were annotated as described above. For SMBCs, we divided the number of SMBCs in each ^13^C-labeled contig pool by the number of protein-coding genes in that pool. We then measured Pearson’s correlation between the genomic feature abundance and each of the activity characteristics averaged across the OTUs that were also ^13^C labeled in each treatment. Within this bulk measurement, a greater percentage of the protein-coding gene pool annotated to a genomic signature can indicate that (i) a greater proportion of the represented genomes contain those genes, (ii) the represented genomes have multiple copies of those genes, or (iii) there is a greater diversity of those genes within the represented genomes. To account for increased false-discovery rate with multiple comparisons, we adjusted *P* values within each activity characteristic using the Benjamini-Hochberg procedure (*n *=* *7).

### Examining genomic signatures of life history strategies in MAGs.

We next assessed associations between genomic features and activity characteristics by comparing the genetic composition of ^13^C-labeled MAGs with the averaged characteristics of the OTUs mapping to those MAGs. As very few 16S rRNA genes were recovered and binned, we matched MAGs to ^13^C-labeled OTUs based on taxonomy and ^13^C-labeling patterns. MAG taxonomy was assigned using GTDB-Tk (66). MAGs were taxonomically mapped to the set of OTUs that matched at the highest corresponding taxonomic level, and then this set of OTUs was filtered to include those that were ^13^C labeled in the same treatment as the MAG. While previous observations indicate that high taxonomic ranks are a poor predictor of life history traits (15), here, we are using taxonomy to match ^13^C MAGs and ^13^C OTUs that are ^13^C labeled in the same sample, by the same substrate, and at the same time. In this way, our MAG-OTU matches have been filtered by function as a result of stable isotope probing, and they are not a random draw from the entire community. This approach leverages isotopic labeling to enhance the functional coherence of MAG-OTU matching while minimizing loss of information due to annotation errors and application of arbitrary sequence cutoffs. Genomic features within the contigs of each MAG were determined as described above, except that for secreted enzymes, Gram-positive or Gram-negative SignalP predictions were assigned based on MAG taxonomy. Gene and SMBC counts were adjusted as before but based on the total protein-coding gene count of the MAGs. We then measured Pearson’s correlation between the genomic feature abundance within the MAGs and each of the activity characteristics averaged across the OTUs mapped to the MAGs. To account for the increased false-discovery rate with multiple comparisons, we adjusted *P* values within each activity characteristic using the Benjamini-Hochberg procedure (*n *=* *8).

### Examining genomic signatures of life history strategies with independent studies.

We analyzed publicly available soil microbiome data sets to determine whether the genomic relationships we observed in ^13^C-labeled MAGs were representative of soil-dwelling bacteria. Seven data sets were chosen, RefSoil (100), Diamond et al. (101), Yu et al. (102), Wilhelm et al. (103), Wilhelm et al. (104), Zhalnina et al. (105), and Li et al. (106). Assemblies from references 101, 102, and 105 were downloaded from GenBank on 21 June 2021 (NCBI accession numbers given in in Data Set S1). Assemblies from 103 and 104 were acquired from the authors. Assemblies from Li et al. (106) were downloaded from figshare (https://figshare.com/s/2a812c513ab14e6c8161). Annotation was performed identically for all assemblies to avoid biases introduced by different annotation pipelines. Protein-coding genes were identified and translated using Prodigal (107) through Prokka (108). Transcription factor genes, SMBCs, and genes encoding transmembrane helices were further annotated as described above. Transporter genes, transcription factor genes, MCP genes, osmotic stress response genes, and SMBCs were identified, and abundances were calculated as described above. 16S rRNA genes and tRNA genes were identified from Prokka annotations. Pearson correlations were analyzed between transporter gene abundances and transcription factor gene abundances, osmotic stress response gene abundances, and SMBC abundances and between the natural log of 16S rRNA gene counts or tRNA gene count MCP gene abundances separately for each independent data set. Within each data set, *P* values were adjusted for multiple comparisons using the Benjamini-Hochberg procedure (*n *=* *4).

### Using trade-offs to define and predict life history strategies.

The C-S-R framework predicts evolutionary trade-offs in energy allocation to resource acquisition across habitats that vary temporally (e.g., variation in disturbance frequency). Since deletion bias in microbial genomes produces streamlined genomes of high coding density, we can assess evolutionary investment in a particular cellular system by quantifying genomic resources devoted to the operation of that system. That is, genetic information must be replicated and repaired with each generation; hence, energy allocation to a given cellular system over evolutionary time can be assessed as the proportion of the genome devoted to that system. To identify putative life history strategies for ^13^C-labeled MAGs, we used *k*-means clustering to group MAG-based genomic investment in transcription factors and resource acquisition. Investment in transcription factors was defined as the transcription factor gene count divided by total gene counts (TF/gene). Relative investment in resource acquisition was determined by summing secreted enzyme and SMBC gene counts, removing duplicates found in both categories, and then dividing by the number of membrane transporter genes [(SE + SM)/MT]. *k*-means clustering was performed using *k*-centroid cluster analysis with the R package flexclust (109) after scaling and centering the two values and using *k *=* *3. Statistical significance was assessed using the Kruskal-Wallis test, and the Dunn test was used to assess *post hoc* comparisons.

We calculated the same trade-offs in genomic investment [TF/gene and (SE + SM)/MT] for RefSoil genomes. Predicted clusters for RefSoil genomes were made using these two genomic signatures as inferred by the R package flexclust (109) and using the three ^13^C-labeled MAG clusters as the training data set. Differences in genomic investments for the eight previously discussed genomic features were then assessed across clusters using the Kruskal-Wallis test with the Dunn test used to assess *post hoc* comparisons. However, in this analysis, adhesion genes were identified as genes with product names containing the terms “adhesion” or “adhesins” because the previously used product names were not found in these annotations.
