# Supplementary material for: Genomic Features Predict Bacterial Life History Strategies in Soil, as Identified by Metagenomic Stable Isotope Probing
Source: mBio. 2023 Mar 6;14(2):e03584-22. doi: 10.1128/mbio.03584-22 (PMC10128055; doi:10.1128/mbio.03584-22)
Supplement: FIG S1 [file mbio.03584-22-s0005.pdf]

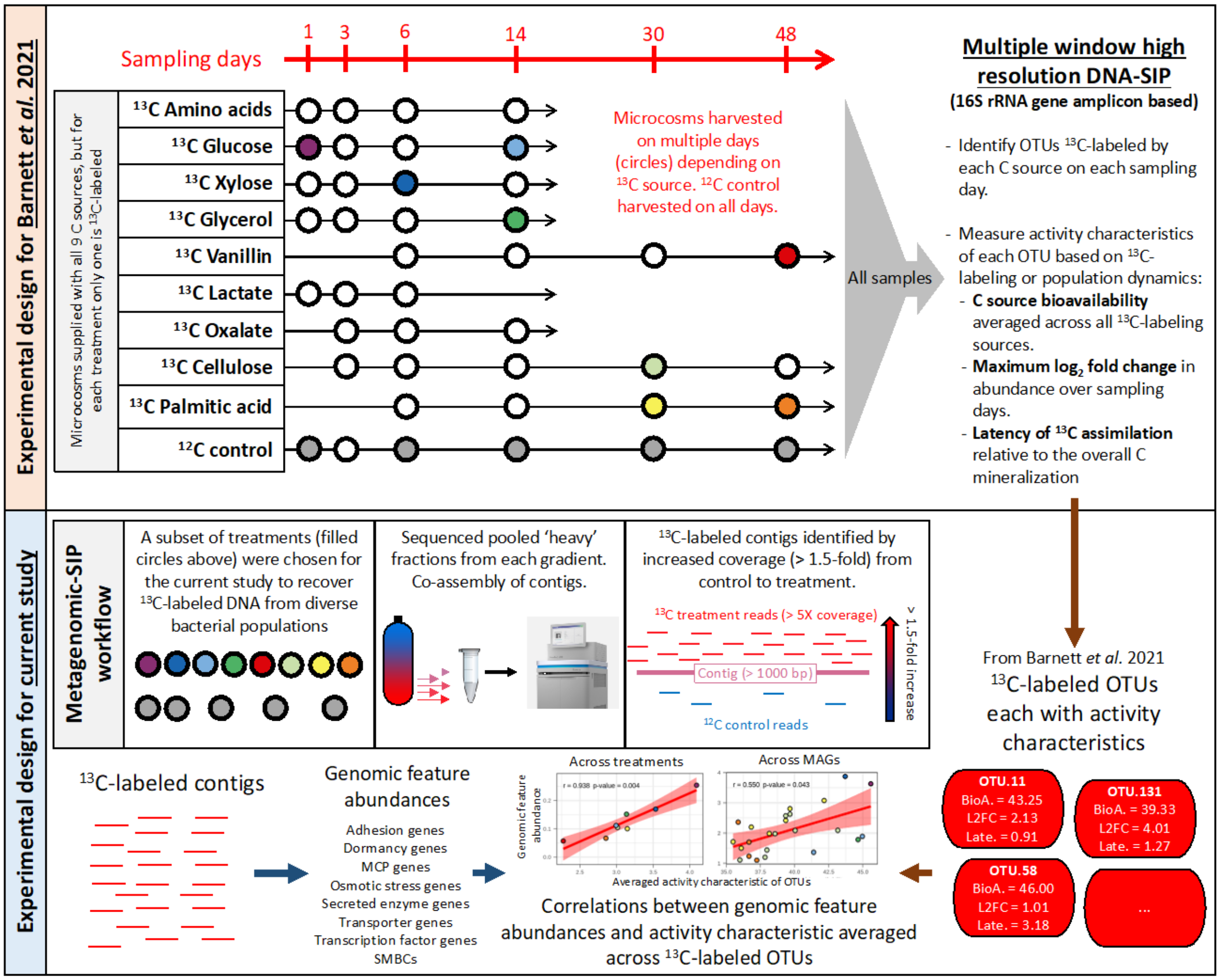

Figure S1: Experimental diagram of the previously described multi-substrate DNA-SIP study from Barnett *et al.*, 2021 (top panel) and the newly described metagenomic-SIP sequencing, processing, and analysis described here (bottom panel). In the top panel, the circles represent the days when microcosms were harvested, while the filled circles represent the samples chosen for metagenomic-SIP sequencing. The original DNA-SIP study used multiple-window high-resolution DNA-SIP to identify bacterial operational taxonomic units (OTU) that assimilated ^{13}C from each of the ^{13}C sources within the harvested microcosms. The ^{13}C-labeling patterns along with population dynamics in soils (unfractionated DNA) were used to generate the three activity characteristics. To compare genomic features in ^{13}C-labeled contigs within a treatment or MAG, we then averaged the characteristics of the OTUs either ^{13}C-labeled in the treatment or taxonomically mapped to the MAG and ^{13}C-labeled in the same treatment respectively. For the metagenomic-SIP sequencing, pooled fractions between 1.72 and 1.77 g ml^{-1} were sequenced. ^{13}C-labeled contigs were distinguished as being > 1000 bp long, having at least 5X coverage in the ^{13}C-treatment library, and having over a 1.5 fold increase in coverage in the ^{13}C-treatment compared to its corresponding ^{12}C-control.
