# Supplementary material for: Genomic Features Predict Bacterial Life History Strategies in Soil, as Identified by Metagenomic Stable Isotope Probing
Source: mBio. 2023 Mar 6;14(2):e03584-22. doi: 10.1128/mbio.03584-22 (PMC10128055; doi:10.1128/mbio.03584-22)
Supplement: FIG S2 [file mbio.03584-22-s0006.pdf]

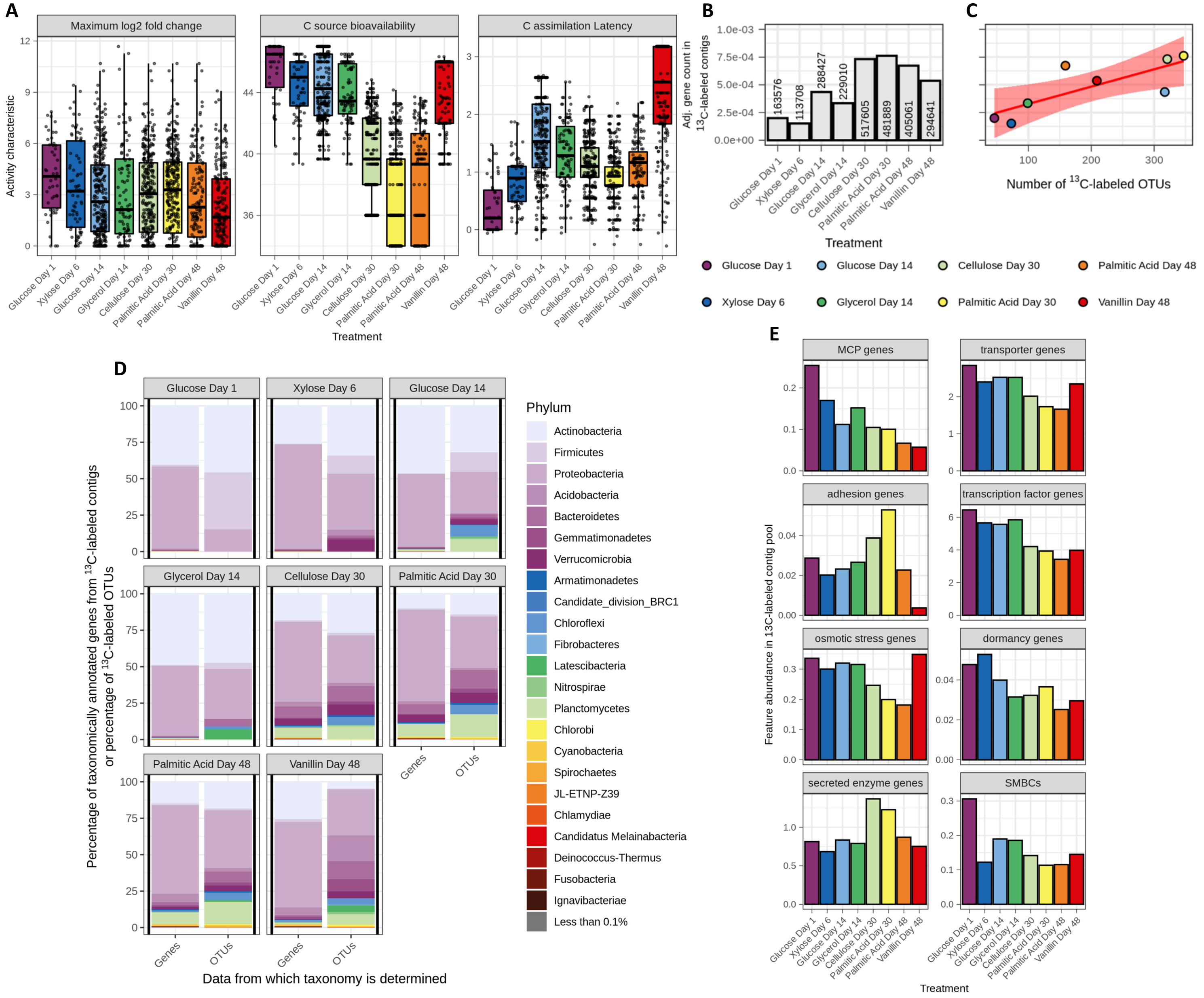

Figure S2: (A) Activity characteristic values of the 13C-labeled OTUs (as previously described in Barnett et al. 2021) detected in each 13C-labeled treatment subjected to metagenomic sequencing. Briefly, maximum log2 fold change represents the change in differential abundance from the initial condition (time zero) to the point when relative abundance was maximal for a given OTU. C source bioavailability is defined as the average bioavailabilty of all the substrates assimilated by each OTU, with the bioavailability of each C source defined operationally based on its mineralization dynamics (as previously described in Barnett et al., 2021). C assimilation latency is defined as the difference in time between the point when 13C source mineralization was maximal and the point at which an OTU was observed to assimilate the 13C-substrate. **(B)** The number of genes in 13C-labeled contigs correlates with the number of 13C-labeled OTUs identified in Barnett et al., 2021, suggesting that we recovered genomic content of the target, active bacteria. Gene count is normalized by the number of reads recovered from the 13C-treatment libraries (**Table S1**). The normalized number of genes found in the 13C-labeled contigs from each treatment. The numbers within or above the bars indicate the number of genes before normalization. **(C)** The relationship between the normalized number of genes in the 13C-labeled contigs and the number of OTUs 13C-labeled under the same treatment (Pearson's r = 0.795, p-value = 0.018). The red line represents the linear regression with red shading indicating the 95% confidence intervals. **(D)** Phylum level breakdown of the taxonomically annotated genes in the 13C-labeled contigs under each treatment (Genes) and the phylum level breakdown of the 13C-labeled OTUs under each treatment in Barnett et al., 2021 (OTUs). Genes used for this analysis were only those with taxonomic annotations. Gene taxonomy was assigned using the IMG pipeline. OTU taxonomy was assigned using the SILVA 111 release. **(E)** Abundance of genes from each feature in the 13C-labeled contigs from each treatment. For all features except SMBCs, feature abundance is calculated as the percentage of total protein coding genes having the feature. For SMBCs, abundance is calculated as the number of SMBCs divided by the total protein coding gene count.
