# Supplementary material for: Genomic Features Predict Bacterial Life History Strategies in Soil, as Identified by Metagenomic Stable Isotope Probing
Source: mBio. 2023 Mar 6;14(2):e03584-22. doi: 10.1128/mbio.03584-22 (PMC10128055; doi:10.1128/mbio.03584-22)
Supplement: TABLE S1 [file mbio.03584-22-s0003.pdf]

Genomic features predict bacterial life history strategies in soil, as identified by metagenomic stable isotope probing

Table S1. Information on the metagenomic-SIP libraries. ^{13}C-labeled contigs were defined as being over 1000 bp long, having at least 5X coverage in the ^{13}C-treatment library and having at least a 1.5 fold increase in coverage between the ^{12}C-control and ^{13}C-treatment libraries after accounting for sequencing depth. The gene counts include all genes predicted from the ^{13}C-labeled contigs in each treatment.

Library	Quality controlled reads	^{13}C-labeled contigs	Genes from ^{13}C-labeled contigs
^{12}C-Control day 1	600,398,782		
^{13}C-Glucose day 1	818,866,782	70,416	163,576
^{12}C-Control day 6	537,771,884		
^{13}C-Xylose day 6	750,644,734	45,590	113,708
^{12}C-Control day 14	778,966,874		
^{13}C-Glucose day 14	662,897,402	120,102	288,427
^{13}C-Glycerol day 14	658,015,110	94,047	229,010
^{12}C-Control day 30	1,279,583,274		
^{13}C-Cellulose day 30	707,180,514	214,068	517,605
^{13}C-Palmitic acid day 30	632,500,566	194,387	481,889
^{12}C-Control day 48	1,326,945,180		
^{13}C-Palmitic acid day 48	602,121,814	157,913	405,061
^{13}C-Vanillin day 48	548,508,008	127,236	294,641
