# Supplementary material for: Genomic Features Predict Bacterial Life History Strategies in Soil, as Identified by Metagenomic Stable Isotope Probing
Source: mBio. 2023 Mar 6;14(2):e03584-22. doi: 10.1128/mbio.03584-22 (PMC10128055; doi:10.1128/mbio.03584-22)
Supplement: TEXT S1 [file mbio.03584-22-s0001.docx]

**Genomic features predict bacterial life history strategies in soil, as identified by metagenomic stable isotope probing**

Samuel E. Barnett, Rob Egan, Brian Foster, Emiley A. Eloe-Fadrosh, Daniel H. Buckley

**Supplemental Results**

*Taxonomy of ^13^C-labeled contigs*

The phylum level breakdown of the taxonomically annotated genes from the ^13^C-labeled contigs differed to some extent from the taxonomy of the ^13^C-labeled OTUs identified in Barnett *et al*. 2021^1^. (Fig. S2c). For example, while *Firmicutes* made up a large portion of the ^13^C-labeled OTUs under glucose day 1, xylose day 6, and glucose day 14, very few genes from ^13^C-labeled contigs were annotated as *Firmicutes* under these treatments. Similarly, *Chloroflexi* OTUs were ^13^C-labeled under cellulose and palmitic acid day 30 and palmitic acid and vanillin day 48, yet few genes annotated to *Chloroflexi* were identified in any ^13^C-labeled contig. There are a number of potential sources for the discrepancy. First, for this metagenomic-SIP experiment we used a simple ‘heavy window’ design while Barnett *et al*. 2021^1^ used multiple-window high-resolution DNA-SIP (MW-HR-SIP). With a heavy window design, DNA from a single pooled buoyant density range is sequenced and coverage of resulting contigs is compared between the ^13^C-treatment and ^12^C-control libraries. In MW-HR-SIP, multiple overlapping heavy buoyant density windows, made up of multiple gradient fractions, are used to compare OTU abundances between a ^13^C-treatment and ^12^C-control gradient. As discussed in Barnett *et al*. 2020^2^, heavy window metagenomic-SIP may exclude low G+C DNA (*e.g., Firmicutes*) because this DNA, even when ^13^C-labeled, may be in exceedingly low abundance within the sequenced heavy window. Heavy window designs may also be less sensitive to ^13^C-labeling for high G+C DNA (*e.g.* *Actinobacteria*) because, even when unlabeled, this DNA may be found in high abundance within the sequenced buoyant density window and therefore no increase in coverage due to isotopic labeling is measured between a ^13^C-treatment library and the ^12^C-control library^2^. We chose our sequenced buoyant density window to reduce these effects but they may still be present. MW-HR-SIP may be less biased in the range of organismal G+C it can capture^3^. There is currently no comparable method to MW-HR-SIP for metagenomic sequencing though gradient resolved SIP^4^ may improve sensitivity to G+C extremes. Second, genome size may influence the apparent relative contributions of each phylum to the gene pools. Genome size can vary widely across all phyla of bacteria^5^ and larger genomes will naturally have many genes, making them appear overrepresented when assessing only genes counts within the gene pool. Third, sequencing bias, either in the 16S rRNA gene sequencing^6^ used by Barnett *et al*. 2021^1^ or in this shotgun metagenomic sequencing (*e.g.* G+C bias)^7^ may affect read recovery from particular bacterial taxa. Fourth, while the JGI IMG pipeline uses a highly diverse and inclusive reference database for metagenome annotations^8^, some taxa may be still be under-represented in the reference, limiting taxonomic annotation of their genes.

*Recovered MAGs of ^13^C-labeled bacteria*

MAGs were binned separately for each treatment-control library pair because treatments represent distinct C sources. Given this binning design, closely related MAGs can share contigs, if these MAGs were ^13^C enriched by multiple C sources (Supplemental Dataset). Since MAGs represent populations, as opposed to individual genomes, the existence of overlapping MAGs allows us to distinguish genetically similar populations that have distinct patterns of C assimilation. We recovered 27 ‘medium quality’ MAGs from the ^13^C-labeled contigs (> 50% completeness and < 10% contamination^9^; Supplemental dataset). Our binning strategy allows MAGs binned from different treatments to share co-assembled contigs. In total, MAGs encompassed 25892 contigs of which 17309 were binned into only one MAG, 7058 were binned into two MAGs, 1523 were binned into three MAGs, and 2 were binned into four MAGs. Contigs shared across MAGs tended to be small, though 210 were over 10000 bp long. We found no MAGs that were completely identical to one another (Supplemental Dataset). There were some cases where a smaller MAG appeared to be a subset of another, with up to 97.3% of its contigs shared with the larger one. When accounting for contig length, the total shared lengths were up to 98.6%. In these cases, however, less than 90% of the larger MAG, both contig and length wise, was shared with the smaller MAG. Overall, 13 MAGs shared over 50% of their contigs and length with another MAG, while 5 MAGs shared over 90% of their contigs and 6 MAGs shared over 90% of their length with other MAGs. Despite these overlaps, we believe that these MAGs likely represent distinct populations based on differences in ^13^C assimilation and growth dynamics.

*Non-statistically significant genomic features*

Three of the eight genomic features we tested using ^13^C contig-based and ^13^C-MAG-based analyses showed no statistically significant correlation to measured activity characteristics: adhesion genes, dormancy genes, and secreted enzyme genes. Further, except for transporter gene counts, all other genomic features were significantly correlated to activity characteristics in contig or MAG data, but not in both datasets. This inconsistency may be due to some features being easier to detect at community level (contig-based) and others being easier to detect at the genome level (MAG-based). A clear example of this is the reduced dynamic range for MCP genes in the MAGs; that is, the MCP count varies little across genomes, but it varies a great deal between metagenomes. In addition, the existence of life history tradeoffs implies that a trait that is beneficial to a group of organisms with one ecological strategy might be detrimental to another set of organisms with a contrasting strategy. If such tradeoffs exist then we expect simple linear correlations to fail and this was the reason that we explicitly looked for evidence of tradeoffs. Consider the case of secreted enzyme gene counts. We see that ruderals have significantly lower secreted enzyme counts than competitors (Fig. 6), though both of these groups otherwise share a range of copiotrophic characteristics (*e.g.*, rapid growth rates, high *rrn* copy number). Simple linear correlations tend clearly resolve copiotrophic traits from oligotrophic traits but they do not capture the tradeoffs that differentiate ruderal strategies from oligotrophic strategies.

Adhesion gene abundance showed no significant relationship to any activity characteristic. Surface adhesion, with the use of adhesin or holdfast proteins, allows bacteria to form tight associations with soil particles. Adhesion is predicted to facilitate degradation of particulate organic matter in soil by allowing for colonization of the particle^4^, similar to its role in colonization of plant roots^10^. The negative trends we observe between adhesion gene abundance and both mean C source bioavailability and mean C assimilation latency (Fig. S2e) follow this prediction. However, since abundance of adhesion genes is very low, even when observing pooled ^13^C-labeled contigs (Fig. S2e), we may be lacking enough resolution to truly measure this feature. Indeed, only a few MAGs had any adhesins or holdfast genes (Fig. S4). In addition, there may exist diverse attachment systems, many of which might be poorly annotated, and this may limit our ability to quantify adhesion in gene annotations. Future studies examining adhesion in relation to C-cycling activity would benefit from a more thorough investigation of adhesion related genes and proteins.

Dormancy is an important set of mechanisms that allow bacteria to survive unfavorable conditions, including nutrient limitation^11,12^. Soil C sources, particularly those of high bioavailability, tend to be transient in soil due to rapid microbial processing, soil interactions, moisture conditions, and input variability. Dormancy is therefore predicted to be a useful strategy for survival during the periods of low C availability^11^. The positive trends we observe between dormancy gene abundance and both C source bioavailability and maximum log_2_ fold change, as well as the negative trend observed to C assimilation latency follow this prediction (Fig. S2e). As with adhesion genes, the set of genes we used to identify dormancy features are not well recovered in either the ^13^C-labeled contigs or MAGs (Fig. S2e, S4). There are numerous dormancy mechanisms in bacteria (*e.g.,* endospores, toxin-antitoxin systems) though many are not well described for a wide variety of taxa^13^. Future work with a greater representation of genomic features of dormancy would greatly improve analyses.

Secreted enzymes are a primary way in which bacteria can break down hard to utilize nutrients such as insoluble particulate organic matter^14–16^. Such C sources must be broken down into smaller molecules in order to be imported and utilized by a bacterium. In soils, these low bioavailability C sources often include plant biomass, necessitating the production of secreted carbohydrate active enzymes to break down structural components such as plant cell walls, proteases to break down protein components into peptides, amino acids and other easily transportable compounds, and lipases to break down membranes and waxes into fatty acids and alcohols. The negative trends we observe between abundance of secreted enzymes and mean C source bioavailability keep with this idea (Fig. S2e, S4).

Finally, we found no genomic feature significantly associated with C assimilation latency, though some predictive trends were observed. We believe that this lack of association was due to the somewhat limited range in latency across the bacterial populations sampled in this study. Across treatments, most ^13^C-labeled OTUs had relatively low latency (Fig. S2). In addition, the OTUs matched to our MAGs had relatively low latency (Fig. S3). No OTUs matched the single MAG recovered from the vanillin day 48 treatment, which would have been most likely to have mapped high latency OTUs. Future studies replicating this experimental design should take this limited latency range into account and target treatments with a greater coverage of C assimilation latencies.

**References**

1. Barnett, S. E., Youngblut, N. D., Koechli, C. N. & Buckley, D. H. Multisubstrate DNA stable isotope probing reveals guild structure of bacteria that mediate soil carbon cycling. *Proc. Natl. Acad. Sci.* **118**, e2115292118 (2021).

2. Barnett, S. E. & Buckley, D. H. Simulating metagenomic stable isotope probing datasets with MetaSIPSim. *BMC Bioinformatics* **21**, 37 (2020).

3. Youngblut, N. D., Barnett, S. E. & Buckley, D. H. SIPSim: A modeling toolkit to predict accuracy and aid design of DNA-SIP experiments. *Front. Microbiol.* **9**, 570 (2018).

4. Wilhelm, R. C., Pepe-Ranney, C., Weisenhorn, P., Lipton, M. & Buckley, D. H. Competitive exclusion and metabolic dependency among microorganisms structure the cellulose economy of an agricultural soil. *MBio* **12**, e03099-20 (2021).

5. Bentley, S. D. & Parkhill, J. Comparative Genomic Structure of Prokaryotes. *Annu. Rev. Genet.* **38**, 771–791 (2004).

6. Tremblay, J. *et al.* Primer and platform effects on 16S rRNA tag sequencing. *Front. Microbiol.* **6**, 771 (2015).

7. Sato, M. P. *et al.* Comparison of the sequencing bias of currently available library preparation kits for Illumina sequencing of bacterial genomes and metagenomes. *DNA Res.* **26**, 391–398 (2019).

8. Chen, I.-M. A. *et al.* IMG/M v.5.0: an integrated data management and comparative analysis system for microbial genomes and microbiomes. *Nucleic Acids Res.* **47**, D666–D677 (2019).

9. Bowers, R. M. *et al.* Minimum information about a single amplified genome (MISAG) and a metagenome-assembled genome (MIMAG) of bacteria and archaea. *Nat. Biotechnol.* **35**, 725–731 (2017).

10. Kolton, M., Frenkel, O., Elad, Y. & Cytryn, E. Potential Role of Flavobacterial Gliding-Motility and Type IX Secretion System Complex in Root Colonization and Plant Defense. *Mol. Plant-Microbe Interact.* **27**, 1005–1013 (2014).

11. Jones, S. E. & Lennon, J. T. Dormancy contributes to the maintenance of microbial diversity. *Proc. Natl. Acad. Sci.* **107**, 5881 LP – 5886 (2010).

12. Kearns, P. J. & Shade, A. Trait-based patterns of microbial dynamics in dormancy potential and heterotrophic strategy: case studies of resource-based and post-press succession. *ISME J.* **12**, 2575–2581 (2018).

13. Dworkin, J. & Shah, I. M. Exit from dormancy in microbial organisms. *Nat. Rev. Microbiol.* **8**, 890–896 (2010).

14. Schimel, J. & Schaeffer, S. Microbial control over carbon cycling in soil. *Front. Microbiol.* **3**, 348 (2012).

15. Sinsabaugh, R. L., Antibus, R. K. & Linkins, A. E. An enzymic approach to the analysis of microbial activity during plant litter decomposition. *Agric. Ecosyst. Environ.* **34**, 43–54 (1991).

16. Ramin, K. I. & Allison, S. D. Bacterial tradeoffs in growth rate and extracellular enzymes. *Front. Microbiol.* **10**, 2956 (2019).
