# Supplementary material for: Genomic Features Predict Bacterial Life History Strategies in Soil, as Identified by Metagenomic Stable Isotope Probing
Source: mBio. 2023 Mar 6;14(2):e03584-22. doi: 10.1128/mbio.03584-22 (PMC10128055; doi:10.1128/mbio.03584-22)
Supplement: FIG S3 [file mbio.03584-22-s0007.pdf]

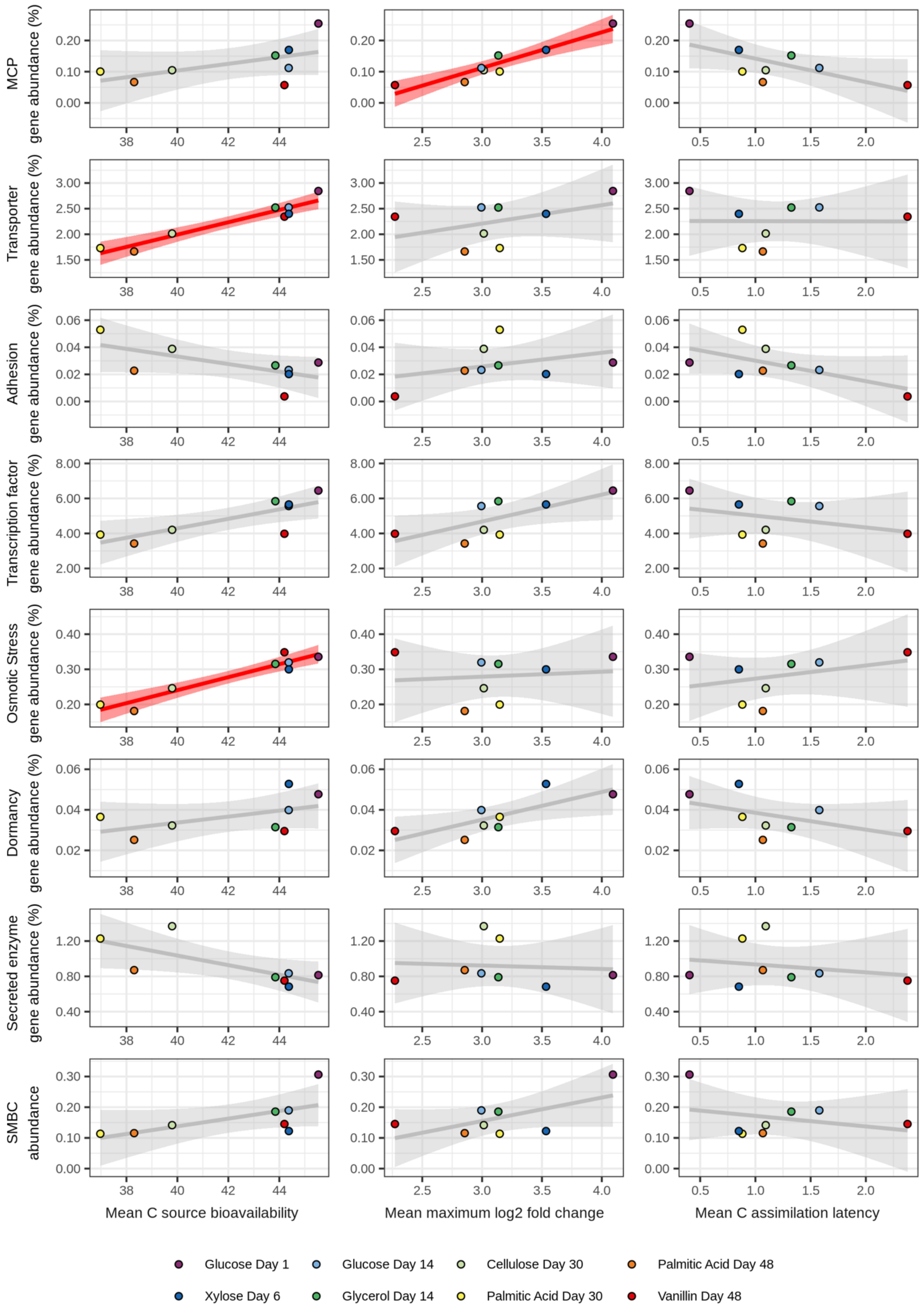

Figure S3: Relationships between the frequency of 8 genome features in ^{13}C-labeled contigs and all three *in situ* activity characteristics of ^{13}C-labeled OTUs across treatments. For all except SMBCs, abundance is calculated as the percent of protein coding genes in ^{13}C-labeled contigs that are annotated within the genomic feature. SMBC abundance is calculated as the SMBC count divided by total protein coding gene count. Red or grey lines represent the linear relationships with shading indicating the 95% confidence intervals. Red relationships are statistically significant, with p-values adjusted for multiple comparisons using the Benjamini-Hochburg procedure (n = 8). Correlation statistics are listed in the Supplemental Dataset.
