# Supplementary material for: Genomic Features Predict Bacterial Life History Strategies in Soil, as Identified by Metagenomic Stable Isotope Probing
Source: mBio. 2023 Mar 6;14(2):e03584-22. doi: 10.1128/mbio.03584-22 (PMC10128055; doi:10.1128/mbio.03584-22)
Supplement: FIG S4 [file mbio.03584-22-s0008.pdf]

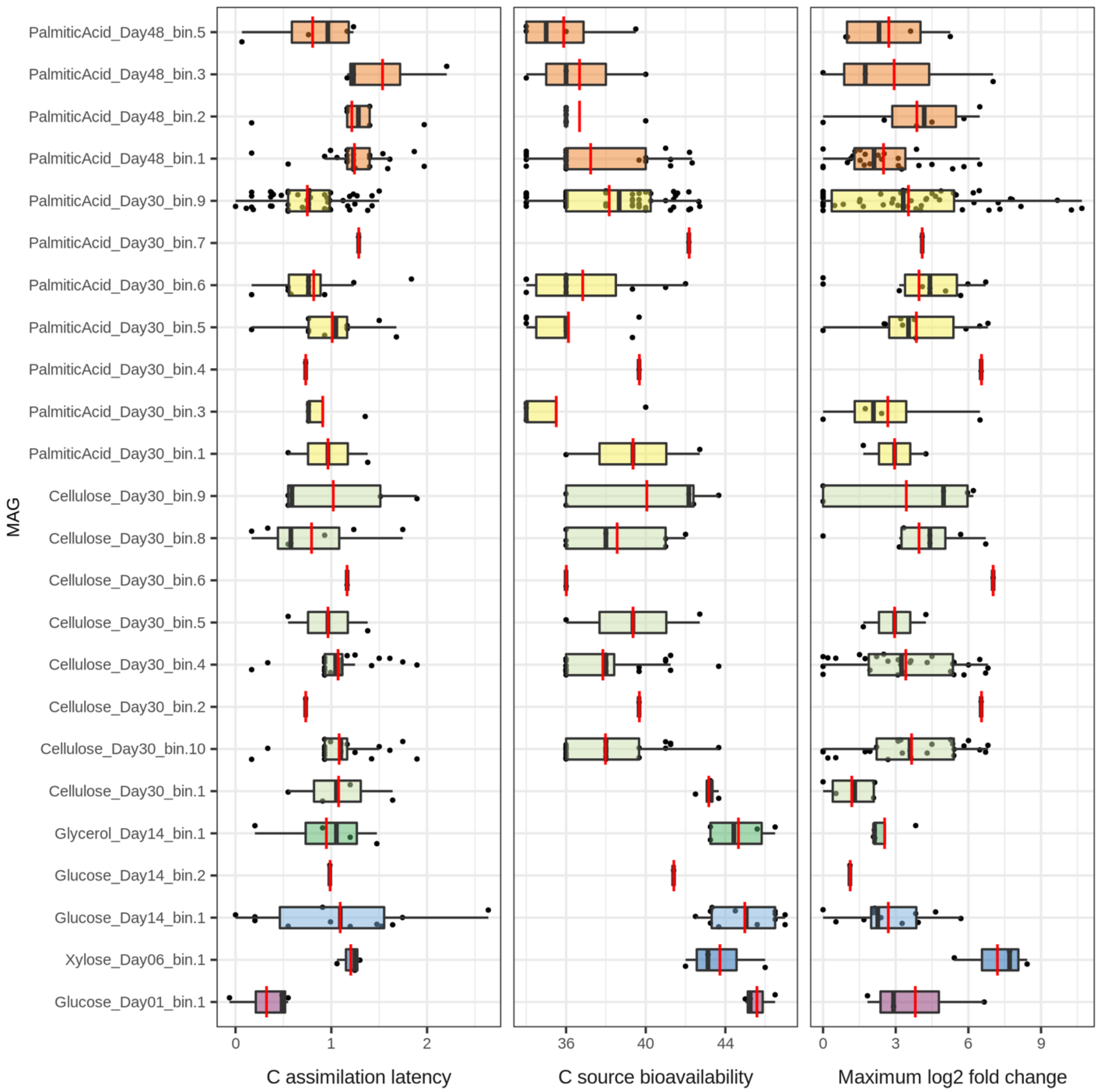

Figure S3: Activity characteristics of the ^{13}C-labeled OTUs mapped to each ^{13}C-labeled MAG. Boxplots are colored by the treatment under which ^{13}C-labeling occurs. Red bars indicate mean values. Three MAGs had no matching OTUs: Cellulose_Day30_bin.7, PalmiticAcid_Day48_bin.4, and Vanillin_Day48_bin.1. MAG and mapped OTU details are found in the Supplemental Dataset.
