# Supplementary material for: Genomic Features Predict Bacterial Life History Strategies in Soil, as Identified by Metagenomic Stable Isotope Probing
Source: mBio. 2023 Mar 6;14(2):e03584-22. doi: 10.1128/mbio.03584-22 (PMC10128055; doi:10.1128/mbio.03584-22)
Supplement: FIG S5 [file mbio.03584-22-s0009.pdf]

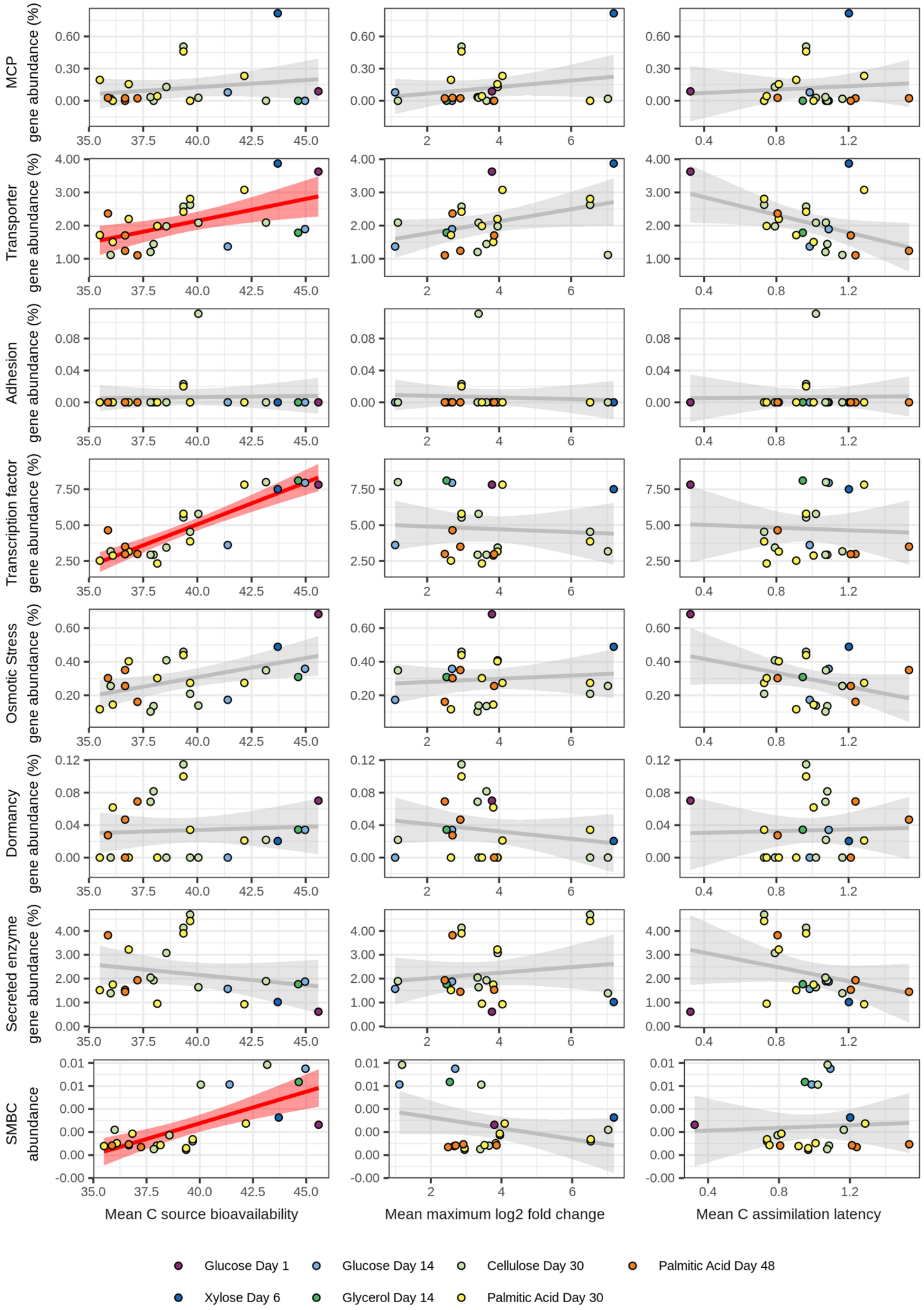

Figure S5: Relationships between abundance of all 8 genome features in ^{13}C-labeled MAGs and all three activity characteristics of OTUs matching MAG taxonomy and ^{13}C-labeling. For all except SMBCs, abundance is calculated as the percent of protein coding genes in the MAG that are annotated within the genomic feature. SMBC abundance is calculated as the SMBC count divided by total protein coding gene count. Red or grey lines represent the linear relationships with shading indicating the 95% confidence intervals. Red relationships are statistically significant, with *p*-values adjusted for multiple comparisons using the Benjamini-Hochburg procedure (n = 8). Correlation statistics are listed in the Supplemental Dataset.
