# Supplementary material for: Genomic Features Predict Bacterial Life History Strategies in Soil, as Identified by Metagenomic Stable Isotope Probing
Source: mBio. 2023 Mar 6;14(2):e03584-22. doi: 10.1128/mbio.03584-22 (PMC10128055; doi:10.1128/mbio.03584-22)
Supplement: TABLE S2 [file mbio.03584-22-s0004.pdf]

**Genomic features predict bacterial life history strategies in soil, as identified by metagenomic stable isotope probing**

**Table S2**. Summary of parameter values for inferred life history clusters. A symbol (+) was added every time a cluster was observed to have a significantly higher value than another cluster in both $^{13}$C-MAG comparisons and the RefSoil comparisons. Abbreviations defined in the text.

| | **MT** | **OS** | **TF** | **MCP** | **Dormancy** | **SMBC** | **SE** | **Adhesion** | ***rrn*** |
|---|---|---|---|---|---|---|---|---|---|
| **Ruderal** | +++[a,b] | +++[a,b] | ++[a,b] | +++[a,b] | ++[b] | | | | |
| **Competitor** | +[b] | ++[a,b] | ++[a,b] | +[b] | | ++[b] | +++[a,b] | ++[a] | |
| **Scarcity tolerant** | | | | | | +[b] | +[b] | | |

[a] support from $^{13}$C-MAG clusters
[b] support from RefSoil clusters
