# Supplementary material for: Genomic Features Predict Bacterial Life History Strategies in Soil, as Identified by Metagenomic Stable Isotope Probing
Source: mBio. 2023 Mar 6;14(2):e03584-22. doi: 10.1128/mbio.03584-22 (PMC10128055; doi:10.1128/mbio.03584-22)
Supplement: FIG S6 [file mbio.03584-22-s0010.pdf]

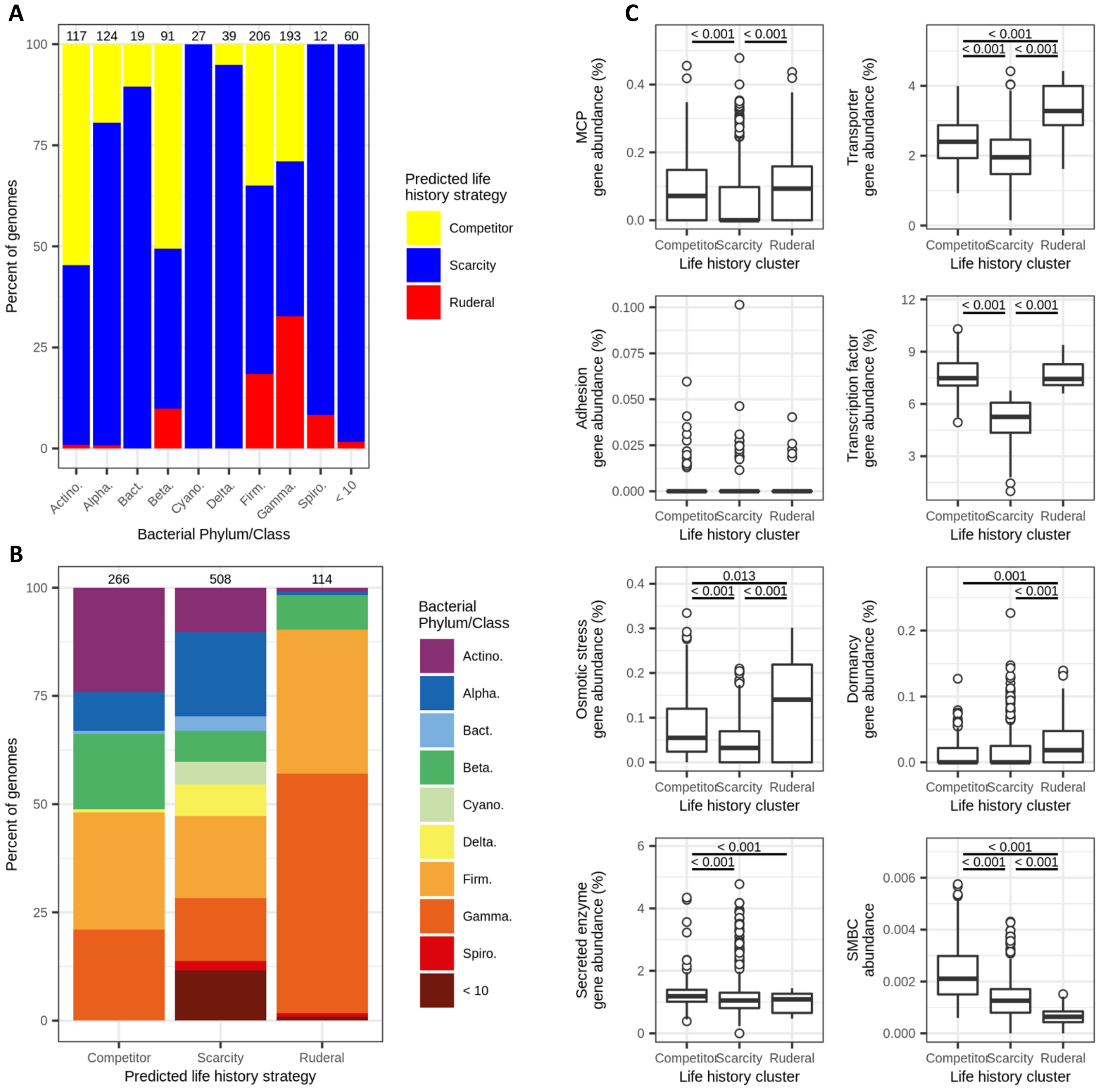

Figure S6: (A) Distribution of RefSoil bacterial taxa (at the phylum or class level) across the life history strategies predicted from clusters of TF:gene and [SE + SM]:MT. Percentage of genomes from each taxa in each predicted life history strategy with total number of genomes used above each stacked bar. **(B)** Percentage of genomes in each predicted life history strategy cluster that are classified to each phylum/-class. Phylum/class abriviations are Actino. = Actinobacteria, Alpha. = Alphaproteobacteria, Bact. = Bacteroidetes, Cyano. = Cyanobacteria, Delta. = Deltaproteobacteria, Firm. = Firmicutes, Gamma. = Gammaproteobacteria, Spiro. = Spirochetes, and ‘< 10’ = taxa that contain less than 10 genomes. **(C)** Genomic investment in gene systems differ across life history strategies predicted from TF:genes and [SE + SM]:MT. Data are from RefSoil genomes with k-means clustering trained by clusters identified from 13C-labeled MAG. In all cases variation across life history clusters was first tested with Kruskal-Wallis tests and where statistically significant (p-value < 0.05) post hoc pairwise tests were performed using Dunn tests.
